# The Costs of Anonymization: Case Study Using Clinical Data

**DOI:** 10.2196/49445

**Published:** 2024-04-24

**Authors:** Lisa Pilgram, Thierry Meurers, Bradley Malin, Elke Schaeffner, Kai-Uwe Eckardt, Fabian Prasser, GCKD Investigators

**Affiliations:** 1 Junior Digital Clinician Scientist Program Biomedical Innovation Academy Berlin Institute of Health at Charité—Universitätsmedizin Berlin Berlin Germany; 2 Department of Nephrology and Medical Intensive Care Charité—Universitätsmedizin Berlin Berlin Germany; 3 Medical Informatics Group Berlin Institute of Health at Charité—Universitätsmedizin Berlin Berlin Germany; 4 Department of Biomedical Informatics Vanderbilt University Medical Center Nashville, TN United States; 5 Institute of Public Health Charité—Universitätsmedizin Berlin Berlin Germany; 6 Department of Nephrology and Hypertension Universitätsklinikum Erlangen, Friedrich-Alexander University Erlangen-Nürnberg Erlangen Germany; 7 See Acknowledgments

**Keywords:** data sharing, anonymization, deidentification, privacy-utility trade-off, privacy-enhancing technologies, medical informatics, privacy, anonymized, security, identification, confidentiality, data science

## Abstract

**Background:**

Sharing data from clinical studies can accelerate scientific progress, improve transparency, and increase the potential for innovation and collaboration. However, privacy concerns remain a barrier to data sharing. Certain concerns, such as reidentification risk, can be addressed through the application of anonymization algorithms, whereby data are altered so that it is no longer reasonably related to a person. Yet, such alterations have the potential to influence the data set’s statistical properties, such that the privacy-utility trade-off must be considered. This has been studied in theory, but evidence based on real-world individual-level clinical data is rare, and anonymization has not broadly been adopted in clinical practice.

**Objective:**

The goal of this study is to contribute to a better understanding of anonymization in the real world by comprehensively evaluating the privacy-utility trade-off of differently anonymized data using data and scientific results from the German Chronic Kidney Disease (GCKD) study.

**Methods:**

The GCKD data set extracted for this study consists of 5217 records and 70 variables. A 2-step procedure was followed to determine which variables constituted reidentification risks. To capture a large portion of the risk-utility space, we decided on risk thresholds ranging from 0.02 to 1. The data were then transformed via generalization and suppression, and the anonymization process was varied using a generic and a use case–specific configuration. To assess the utility of the anonymized GCKD data, general-purpose metrics (ie, data granularity and entropy), as well as use case–specific metrics (ie, reproducibility), were applied. Reproducibility was assessed by measuring the overlap of the 95% CI lengths between anonymized and original results.

**Results:**

Reproducibility measured by 95% CI overlap was higher than utility obtained from general-purpose metrics. For example, granularity varied between 68.2% and 87.6%, and entropy varied between 25.5% and 46.2%, whereas the average 95% CI overlap was above 90% for all risk thresholds applied. A nonoverlapping 95% CI was detected in 6 estimates across all analyses, but the overwhelming majority of estimates exhibited an overlap over 50%. The use case–specific configuration outperformed the generic one in terms of actual utility (ie, reproducibility) at the same level of privacy.

**Conclusions:**

Our results illustrate the challenges that anonymization faces when aiming to support multiple likely and possibly competing uses, while use case–specific anonymization can provide greater utility. This aspect should be taken into account when evaluating the associated costs of anonymized data and attempting to maintain sufficiently high levels of privacy for anonymized data.

**Trial Registration:**

German Clinical Trials Register DRKS00003971; https://drks.de/search/en/trial/DRKS00003971

**International Registered Report Identifier (IRRID):**

RR2-10.1093/ndt/gfr456

## Introduction

Sharing data from clinical studies can accelerate scientific progress, improve transparency, and increase the potential for innovation and collaboration [1]. Scientific data sharing has been encouraged by a range of regulatory agencies [2] and is required by many scientific journals [3]. However, there are various challenges to realizing data sharing in practice. For example, the data should satisfy FAIR (findable, accessible, interoperable, and reusable) principles [4], while sharing policies need to comply with relevant privacy laws, such as the European General Data Protection Regulation [5]. Uncertainty in handling personal data is one of the major challenges to collaborative research [6-9].

Privacy-enhancing technologies, including anonymization algorithms, can maintain the privacy of study participants when sharing data [10,11]. Anonymization reduces privacy risks by altering data in a manner such that it is highly unlikely that it can be related to a person. Anonymization can be performed using various transformation mechanisms, such as suppression, randomization, or generalization. Software-enabled solutions have been developed with implementations of published algorithms to support this process [12]. Yet, there is an inherent trade-off between the reduction of privacy risks and the utility of the data that can be shared [13]. In this respect, a key concern is that the amendments needed to maintain privacy at a certain level may adversely influence the inherent statistical properties of the data.

This challenge has been studied extensively in theory [14], and the evidence for utility-preserving anonymization is growing [12,15-18]. However, anonymization has not been broadly adopted in clinical practice. Multiple studies report substantial gaps in data availability and stress the lack of practical guidance [8,9,19-22]. The need for a better understanding is also supported by a review that found most reported successful disclosure attacks on anonymized data were enabled by incorrectly applying anonymization algorithms [23]. In addition, while many approaches have been developed for capturing and reducing privacy risks, these are typically evaluated using general-purpose utility measures and only rarely real-world individual-level clinical data, providing little insights into their performance in real-world applications [21,22,24]. Metrics based on such applications are comparatively less reported [15,25-28], yet greatly needed to gain a better understanding of privacy-utility trade-offs as well as to provide targeted recommendations for data providers.

In this study, we aim to provide a better understanding of the opportunities for sharing individual-level data from clinical studies. Specifically, we investigate how different anonymization algorithms affect the utility in a real-world application using data and scientific results from the German Chronic Kidney Disease (GCKD) study [29].

## Methods

### Data and Real-World Application

The GCKD study is a nationwide prospective observational cohort to study the natural course of chronic kidney disease (CKD) and to better understand associations between patient characteristics and disease progression [29]. More than 150 outpatient and 11 university-hospital study sites contributed to the recruitment of 5217 patients between March 2010 and March 2012 and subsequent follow-up. Data collection resulted in a high-dimensional data set of more than 4000 variables.

To assess the utility of anonymized data in a real-world application, we studied its performance in downstream analyses. These aimed at describing the disease burden and risk profile of patients with CKD at baseline as previously published by Titze et al [30]. The variables relevant for this application were selected, aggregated, and calculated through multiple preprocessing steps (Figure S1 in Multimedia Appendix 1). The final curated data set was composed of 70 variables and referred to as original for the remainder of this paper.

### Threat Model

Based on the assumption that the data will be disclosed through some web-based platform for sharing data from clinical studies [31-33] with additional measures of control in place (ie, data use agreements and compatible legal environment), we assumed a controlled access scenario [34]. In this context, the aim of anonymization was to provide safeguards in case of an accidental disclosure, for example, a breach of the recipient’s local security measures [35].

In line with guidelines and recommendations on clinical trial data sharing, we focused on protecting the data from linkage and recognition of subject identities (ie, reidentification) [10]. To detect variables that could be used for reidentifying study participants, a 2-step procedure was adopted. First, a qualitative risk assessment was performed based on international guidelines that document lists of potentially linkable variables [36-39]. Next, a semiquantitative risk assessment was performed by studying the variables’ availability (variables likely to be known to adversaries), replicability (variables that occur repeatedly in relationship to the individual), and distinguishability (variables that make, alone or in combination, individuals unique) [40]. This method has been successfully applied for several real-world data sets [41-43]. The scoring system was adapted to our real-world application according to literature and expert knowledge. In brief, availability, replicability, and distinguishability were quantified from low (1) to high (3), and the sum was calculated as the score per variable. A score of greater than 5 was applied as a threshold for the recognition of a “risky” variable. Overall, we determined that 6 of the 70 variables needed to be protected against reidentification: age, gender, height, weight, BMI, and history of renal biopsy. The underlying reasons and results of the 2-step procedure are provided in Table S1 in Multimedia Appendix 1 [40,44].

We further screened for interdependent relationships between variables in the data set (eg, height, weight, and BMI). Transforming them independently can result in 2 issues. First, the transformed values may no longer be logically consistent. Second, back-calculation may narrow down intended generalization intervals and leak information that undermines established risk thresholds. Among our variables, this was true for the anthropometric data height, weight, and BMI. To account for this, we removed either height and weight or BMI from the data depending on the configuration scenario (see Data Transformation section).

### Reidentification Risk Assessment and Thresholds

Following guidelines for clinical trial data anonymization, we quantified and reduced reidentification risks according to probabilistic prosecutor risk (PR) and marketer risk (MR) models [38]. The prosecutor model provides risk estimates under the assumption that the data recipient attempts reidentification against a specific record for which he or she already knows its membership in the data set. Protecting against such attacks also protects the data from reidentification by less knowledgeable data recipients who do not have prior knowledge about membership. By contrast, the marketer model provides an estimate of the average success probability that can be expected of such a less knowledgeable data recipient. Both risk estimates can be calculated from the distinguishability of records in the data set regarding the risky variables. Let *u*(*r*) be the number of records indistinguishable from a record *r* regarding the risky variables (including *r* itself). Then, the risk of each record is 1/*u*(*r*). Data set–level risk estimates can be derived from the distribution of the risks of all records, with the PR referring to the maximum and MR to the average of this distribution [45]. A data set can then be protected from reidentification by transforming it in a way that those risk estimates fall below given thresholds. The privacy model aiming at the PR is typically called *k*-anonymity, whereas the privacy model that addresses a combined view of the PR and MR is called strict-average risk [38]. We studied anonymized data sets with PR and MR thresholds ranging from 1 to 0.02, respectively.

In our analysis, we put a special focus on three risk thresholds: (1) 0.5 (ie, a group size of 2), as this is the greatest risk smaller than 1 that can be measured in approaches built upon distinguishability; (2) 0.09 (ie, a group size of 11), as this is a threshold that has been recommended for sharing data from clinical studies [42,43]; and (3) 0.03 (ie, a group size of 33), as this is the smallest threshold that could be enforced without resulting in additional variables being fully censored in the anonymization process. From these thresholds, 4 privacy levels from moderate to very strict that we highlight in our analyses were derived. We denoted the privacy levels as percentages: (1) 50% PR combined with 9.09% MR, denoted 50% PR+9.09% MR, (2) 50% PR+3.03% MR, (3) 9.09% PR, and (4) 3.03% PR.

### Data Transformation

The anonymized data sets were realized by generalizing and suppressing variables using the open-source tool ARX (Institute of Medical Informatics, Statistics and Epidemiology at Technical University of Munich and Medical Informatics Group at the Berlin Institute of Health, Charité—Universitätsmedizin Berlin) [46]. Generalization categorized continuous data into intervals of different sizes (hierarchies) to prevent distinguishability. Its configuration included the definition of hierarchies, grouping factors, and maximum and minimum values (Table 1). Values of 1 variable were transformed consistently (ie, to the same hierarchy level). We chose this process because it simplifies downstream statistical analyses. According to generally accepted rates of missing data for statistical analyses, an overall limit of 10% on the number of records that could be suppressed was specified [47,48]. In the dichotomous variables (ie, gender and renal biopsy), only suppression was applied.

Two different configurations were investigated: (1) a generic scenario that aims to support multiple general medical uses without restriction in the generalization hierarchies applied and (2) a use case–specific scenario in which generalization was restricted in variables that were important for our real-world application [30]. Different strategies were also followed to account for the interdependent relationship between the anthropometric data. In the generic scenario, we transformed height and weight and removed BMI from the data to simulate a situation where it was unknown if BMI would be of relevance to the study. In the use case–specific scenario, we took the relevance of BMI into account and removed height and weight from the data but preserved BMI. Table 1 illustrates the characteristics of the 2 scenarios. In total, we created 200 anonymized data sets based on 100 different risk thresholds in 2 configuration scenarios (Figure S1 in Multimedia Appendix 1).

**Table 1:** Differences in generalization between the generic and the use case–specific scenario.^a^

	Generic scenario			Use case–specific scenario	
	Minimum-maximum value	Maximum generalization	Minimum-maximum value		Maximum generalization
Age (years)	15-80	Not defined	18-80		10-year intervals
Height (cm)	20-280	Not defined	Removed		Removed
Weight (kg)	0-160	Not defined	Removed		Removed
BMI (kg/m^2^)	Removed	Removed	15-70		<25.0^b^; 25.0-29.9^c^; ≥30.0^d^

^a^To account for collinearity, we generalized height and weight and removed BMI in the generic scenario. In the use case–specific scenario, BMI was generalized, and height and weight were removed. In this scenario, explicit minimum and maximum values were extracted from the original data set, and generalization was restricted in variables that were relevant to our real-world application. The hierarchies have been archived on the web [49].

^b^Underweight or normal weight.

^c^Overweight.

^d^Obesity.

### Privacy Assessment of Anonymized Data

Due to the consistent transformation of variables, predefined thresholds did not necessarily translate into the actual risk. We therefore calculated the empirical PR and MR and screened for differences from our predefined thresholds (ie, overprotection). In our figures, we present privacy as a spectrum from 1–maximum PR to 1–average PR (ie, MR) and 1–minimum PR. Apart from maximum PR, we chose to include average and minimum PR in our assessment. Considering the potential to overestimate reidentification risks [23], the average and minimum PR represent important additional guiding factors when implementing real-world anonymization algorithms [43].

### Utility Assessment of Anonymized Data

We analyzed general-purpose (ie, generic) utility of the anonymized data sets as well as the degree to which they could be relied upon to reproduce results from the original data describing the disease burden and risk profile of patients with CKD at baseline (use case–specific utility) [30].

To determine general-purpose utility (1) at the cell level, we measured the granularity of the variables in the data set and (2) on the variable level, we applied the nonuniform entropy model that measures deviations in variable distributions. Both were compiled into data set–level measures by averaging their results across all records or all variables, respectively [50-52]. All results were normalized into the range of 0% (all information removed) to 100% (no information modified at all).

To evaluate use case–specific utility, all analyses were performed on the original and the 200 anonymized data sets. To measure reproducibility, we made use of the estimate agreement as described in the context of real-world evidence versus randomized controlled trials [53]. As an estimate agreement, we introduced the relative overlap in 95% CI lengths of the numbers and percentages between the original and anonymized data sets. For this purpose, the proportion or mean 95% CI was determined by the Wilson score interval and 2-tailed *t* test, respectively, and the 95% CI lengths in the anonymized data sets were compared to those in the original data set as proposed by Karr et al [24]. We compiled the relative overlap in 95% CI lengths into table-level measures by averaging all table cells and into data set–level measures (overall average 95% CI overlap) by averaging all analyses including the ones covering only variables that were not affected by the anonymization procedure.

The use case–specific metrics based on 95% CI overlap neglected variables with scale transformation through the anonymization process (ie, age, height, weight, and BMI). In these variables, we compared resulting hierarchy levels and presented their effect on the results visually.

### Technical Implementation

ARX (version 3.9.1; published November 2022) was used for anonymization of the data. The data management, analyses, and visualizations were performed using R (version 4.1.0; R Foundation for Statistical Computing), Python (version 3.11; Python Software Foundation), and built-in functions of ARX for general-purpose metrics and the risk models [46].

### Ethical Considerations

All methods were carried out in accordance with the Declaration of Helsinki. The GCKD study was approved by local ethics committees (Friedrich-Alexander University Erlangen-Nürnberg, Germany, 3831) and registered in the national registry for clinical studies (DRKS 00003971). Informed consent was obtained from all participants prior to enrollment. The participants did not receive any form of compensation. Approval for this study was covered by the approval of the ethics committees of the GCKD study. Participants’ data are stored in pseudonymized form in the study database. The database is on a server at the Regional Computer Centre of the University Hospital in Erlangen. All aspects of data backup and security are based on relevant guidelines and in accordance with the German Federal Data Protection Act.

## Results

### Empirical Residual Risks

Prior to anonymization, 5112 (98%) records in the original data set were unique regarding the variables that could be used for reidentification. Table 2 presents the empirical PR and MR after having transformed the data set considering different risk thresholds. As can be seen, the process of consistent transformation often resulted in overprotection, with minimal and average PR (ie, MR) being below the specified thresholds.

**Table 2:** Empirical minimum, average, and maximum prosecutor risk (PR) and marketer risk (MR).^a^

	Generic scenario			Use case–specific scenario		
	Maximum PR, %	Average PR, % (ie, MR)	Minimum PR, %	Maximum PR, %	Average PR, % (ie, MR)	Minimum PR, %
50% PR+9.09% MR	50	8.7	0.7	50	6.9	0.5
50% PR+3.03% MR	33.3	3	0.2	50	2.5	0.3
9.09% PR	9.1	1.4	0.1	9.1	1.6	0.3
3.03% PR	2.9	0.6	0.1	3	0.9	0.2

^a^We report results for the following four risk thresholds: (1) 50% PR+9.09% MR, (2) 50% PR+3.03% MR, (3) 9.09% PR, and (4) 3.03% PR. It can be seen that empirical risks can be lower than the specified risk thresholds due to consistent data transformation (ie, overgeneralization).

### Privacy-Utility Trade-Off

**Figure 1:**
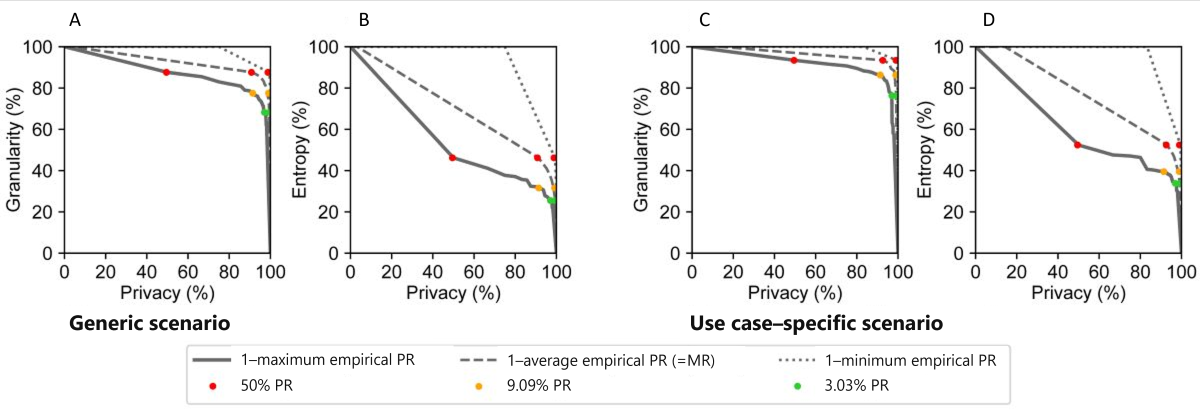
Privacy-utility curves based on general-purpose utility metrics. Granularity and nonuniform entropy served as general-purpose utility metrics. Privacy is demonstrated as 1–empirical maximum PR, average PR (ie, MR), and minimum PR. We used the anonymization processes implementing thresholds on PR for generating the points on the curve: 50% PR, 9.09% PR, and 3.03% PR. Results of granularity in the (A) generic and (C) use case–specific anonymized data sets and results of entropy in the (B) generic and (D) use case–specific anonymized data sets are shown. Results for 50% PR+MR were analogous and are illustrated in Figure S2 in Multimedia Appendix 1. The extreme points at (0,100) and (100,0) have been added to the graph but were not directly measured. MR: marketer risk; PR: prosecutor risk.

**Figure 2:**
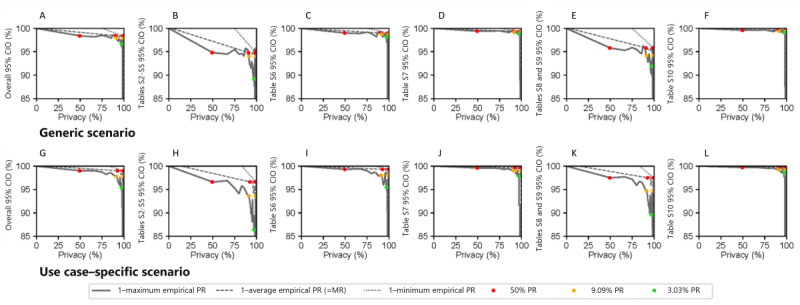
Privacy-utility curves using use case–specific utility metrics based on 95% CIO. 95% CIO was calculated on the data set level (overall 95% CIO) and analysis level. Two analyses (glomerular filtration rate and albuminuria categories and comparison of estimated glomerular filtration rate equations) were not affected by anonymization at all (100% overlap) and are therefore not displayed separately. Privacy is demonstrated as 1–maximum PR, average PR (ie, MR), and minimum PR. We used the anonymization processes implementing thresholds on PR for generating the points on the curve: 50% PR, 9.09% PR, and 3.03% PR. Results of the overall 95% CIO in the (A) generic and (G) use case–specific anonymized data sets and results of the 95% CIOs on analysis level in the (B-F) generic and (H-L) use case–specific anonymized data sets are shown. Results at the estimate level are shown in Tables S2-S10 in Multimedia Appendix 1. Results for 50% PR+MR were analogous and are illustrated in Figure S3 in Multimedia Appendix 1. The extreme points at (0,100) and (100,0) have been added to the graph and were not directly measured. CIO: CI overlap; MR: marketer risk; PR: prosecutor risk.

Next, we studied how well the anonymization approach enabled trading off data privacy against utility. Figure 1 presents privacy-utility trade-off curves when scaling risk thresholds for PR against granularity and entropy as general-purpose utility metrics. Privacy was calculated as 1–empirical PR for minimum, average (ie, MR), and maximum PR, respectively. Figure 2 presents analogous curves for use case–specific utility metrics (ie, 95% CI overlap). When scaling risk thresholds for MR in 50% PR+MR, similar results were observed, such that they are deferred to Figures S2 and S3 in Multimedia Appendix 1.

As can be seen from the results shown in Figure 1, the curve is flatter between the 50% PR and 9.09% PR threshold than between 9.09% PR and 3.03% PR. Thus, a gain in privacy was accompanied by a comparatively lower loss in utility across this risk-utility space. At lower privacy levels than the 50% PR threshold, a high initial loss was observed in entropy but not in granularity. It can be seen from these results that the process had a nontrivial impact on variable distributions, pushing it toward a greater amount of privacy than (general-purpose) utility. For example, in the generic scenario, granularity varied between 87.6% (50% PR+9.09% MR) and 68.2% (3.03% PR), while entropy was generally lower with estimates between 46.2% and 25.5%, respectively.

The use case–specific utility is presented as an overall 95% CI overlap and as an 95% CI overlap on the analysis level in Figure 2 when scaling thresholds for PR and in Figure S3 in Multimedia Appendix 1 for 50% PR+MR. Compared to Figure 1 (general-purpose utility), privacy gain could be achieved with a minor impact on utility in this case. The overall 95% CI overlap in the generic scenario varied from 98.4% at 50% PR+9.09% MR to 96.7% at 3.03% PR.

**Figure 3:**
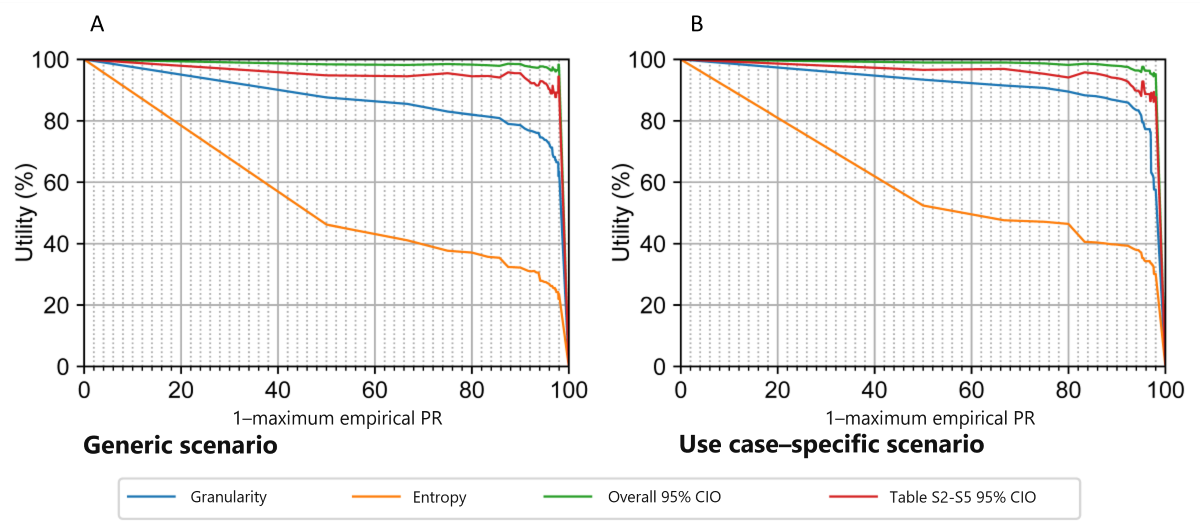
Generic and use case–specific utility metrics. Calculated utility metrics are illustrated in comparison. Granularity and nonuniform entropy served as general-purpose utility metrics. 95% CIO was calculated on the data set level (overall 95% CIO) and analysis level. The latter is exemplary illustrated for the main analysis (Tables S2-S5 in Multimedia Appendix 1, 95% CIO). 95% CIO excluded variables with scale transformation. Privacy is illustrated as 1–maximum PR. We calculated metrics in (A) generic and (B) use case–specific anonymized data sets. Results for 50% PR+MR were analogous and can be drawn from the privacy-utility curves in Figures S2 and S3 in Multimedia Appendix 1. The extreme points at (0,100) and (100,0) have been added to the graph and were not directly measured. CIO: CI overlap; PR: prosecutor risk.

Figure 3 illustrates the differences between the applied utility metrics to point out the multidimensionality of utility. In our real-world application, results from use case–specific metrics were consistently above the ones from general-purpose metrics.

### Reproducibility of Prior Study Results

**Figure 4:**
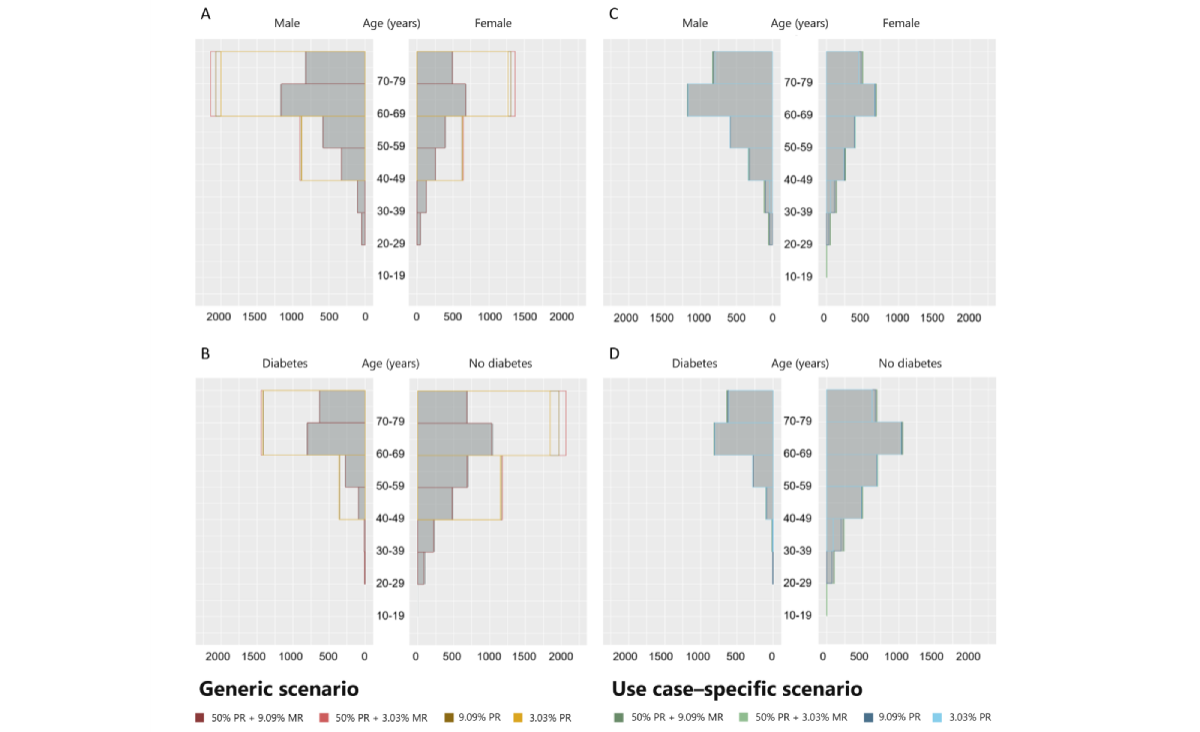
Age distribution stratified by gender and the presence of diabetes mellitus in the original and anonymized data sets. Anonymization was applied as defined in the (A and B) generic and (C and D) use case–specific scenario. Bar plots illustrate counts for anonymized data at selected privacy level: 9.09% MR+50% PR, 3.03% MR+50% PR, 9.09% PR, and 3.03% PR. The figure derived from the original data is illustrated in gray. MR: marketer risk; PR: prosecutor risk.

A more detailed analysis of reproducibility was performed by comparing analyses on estimate level for the selected privacy levels: (1) 50% PR+9.09% MR, (2) 50% PR+3.03% MR, (3) 9.09% PR, and (4) 3.03% PR. We conducted 7 analyses to describe the disease burden and risk profile of patients with CKD at baseline: the disease burden and risk profile stratified by gender and presence of diabetes mellitus (main results, Tables S2-S5 in Multimedia Appendix 1 [24]), characteristics stratified by inclusion criteria (Table S6 in Multimedia Appendix 1), biopsy rates per leading cause (Table S7 in Multimedia Appendix 1), cardiovascular disease burden stratified by gender and the presence of diabetes mellitus (Tables S8 and S9 in Multimedia Appendix 1), the characteristics stratified by diabetes mellitus and diabetic nephropathy (Table S10 in Multimedia Appendix 1), the distribution of glomerular filtration rate and albuminuria categories, and the comparison of estimated glomerular filtration rate (eGFR) equations. The last 2 analyses are not shown as they only covered variables that were not affected by the anonymization procedure resulting in a 100% 95% CI overlap. Additional information on the presumed cause of CKD, patient awareness, and the age distribution of patients stratified by gender and the presence of diabetes mellitus was calculated and illustrated as figures. While no variable was affected by the anonymization process in the first 2 analyses (results not shown), the effects of anonymization for the last one are depicted in Figure 4.

**Figure 5:**
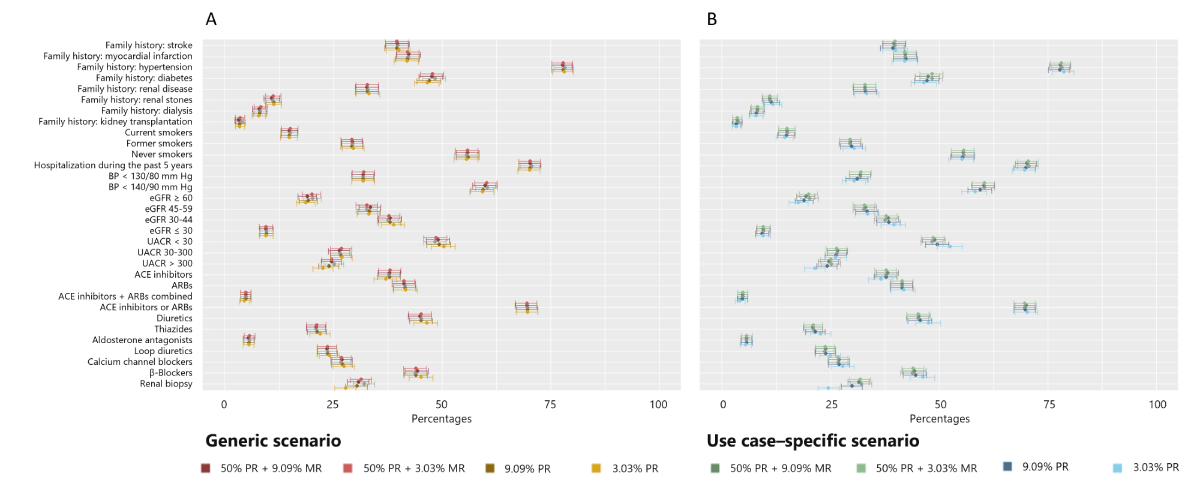
Proportion, CIs, and overlap in the interval lengths for descriptive analyses of the subset of female participants who did not have diabetes. Anonymization was applied as defined in the (A) generic and (B) use case–specific scenario. Results are shown for selected privacy levels: 9.09% MR+50% PR, 3.03% MR+50% PR, 9.09% PR, and 3.03% PR. Only categorical parameters are presented as percentages referred to the numbers excluding missing with proportion 95% CI. 95% CI for both original and anonymized data were calculated based on the Wilson score interval and are displayed in the figure. For the original data, 95% CI is illustrated in gray, and for anonymized data, colors can be depicted from the legend. ACE: angiotensin-converting enzyme; ARBs: Angiotensin II receptor blockers; BP: blood pressure; eGFR: estimated glomerular filtration rate; MR: marketer risk; PR: prosecutor risk; UACR: urine albumin-to-creatinine ratio.

We focused on the reproducibility of the main results (Tables S2-S5 in Multimedia Appendix 1). This included the 95% CI overlap and whether the result of anonymized data was within the original 95% CI (Figure 5 and Tables S2-S5 in Multimedia Appendix 1). The main results were stratified by gender and the presence of diabetes mellitus. For the subset of female participants who did not have diabetes, the results of the 95% CI overlaps at estimate level are shown in Tables S2 and S3 in Multimedia Appendix 1 as well as Figure 5. The original data set included 1462 female participants who did not have diabetes. Due to suppression in the anonymization process, the number decreased at (1) 50% PR+9.09% MR, (2) 50% PR+3.03% MR, (3) 9.09% PR, and (4) 3.03% PR to 1385, 1407, 1360, and 1309, respectively, in the generic scenario and to 1414, 1451, 1342, and 1218, respectively, in the use case–specific scenario. We detected modestly overlapping 95% CIs (<50%) in estimates of the variables renal biopsy (12.4%), urine albumin-to-creatinine ratio (UACR)>300 mg/g (46.2%), and eGFR (47.4%) at 3.03% PR in the generic scenario. In the use case–specific scenario, this was true for estimates of the variables UACR>300 mg/g (19.6%), UACR<30 mg/g (23.9%), eGFR≥60 mL/min (41.6%), eGFR (21.6%), and systolic blood pressure (41.7%) at 3.03% PR. At this privacy level, there was also a nonoverlapping 95% CI (0%) measured for renal biopsy (95% CI 22.2-27.1 vs 29.7-34.5). The modest and nonoverlapping 95% CIs were accompanied by results that were not within the original 95% CI. At lower privacy levels, there were no such constraints. When looking at the other subsets (Tables S4 and S5 in Multimedia Appendix 1), the 95% CI overlap at the estimate level revealed similar results with sporadic modestly (n=7) and nonoverlapping (n=3) 95% CIs.

Considering all analyses, the main results and the results on cardiovascular disease burden were most influenced by a lower 95% CI overlap (Figure 2B, E, H, and K). This is most likely due to the stratification by the variable gender and the large amount of further modified variables in these analyses. Completely overlapping 95% CIs (100%) were reached for 2 of the 7 analyses (glomerular filtration rate and albuminuria categories and comparison of eGFR equations) and for 2 of the 3 figures (presumed cause of CKD and patient awareness). In these analyses, there was no variable affected by the anonymization process, and the results are therefore not shown separately. The trends of affected tables (Tables S6-S10 in Multimedia Appendix 1) at the estimate level were similar to the main results. Two analyses (Tables S7-S9 in Multimedia Appendix 1) could be replicated without modestly overlapping 95% CIs. Within the other tables, estimates exhibited sporadic modestly (n=11) and nonoverlapping (n=2) 95% CIs but by far the majority of estimates exhibited a 95% CI overlap of over 50% across all privacy levels.

**Figure 6:**
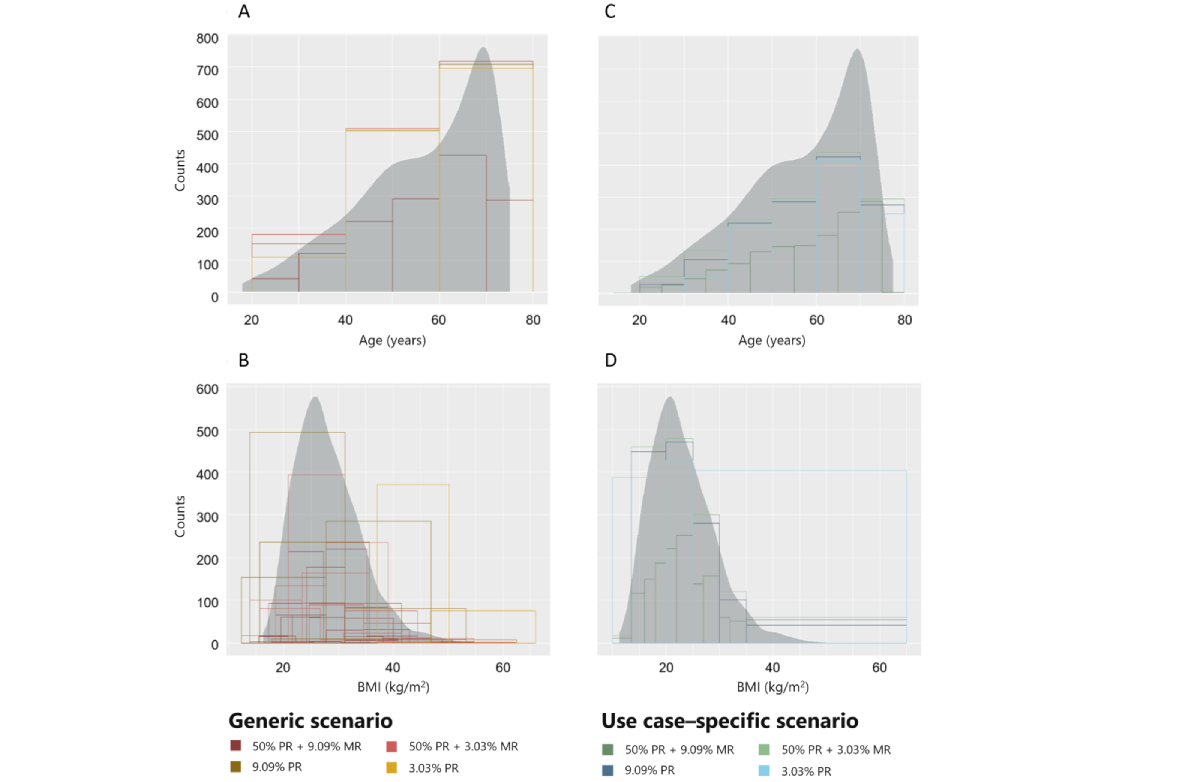
Illustration of age and BMI of female participants who did not have diabetes in the original and anonymized data sets. Anonymization was applied as defined in the (A and B) generic and (C and D) use case–specific scenario. Bar plots illustrate counts for anonymized data at selected privacy level: 9.09% MR+50% PR, 3.03% MR+50% PR, 9.09% PR, and 3.03% PR. The original data are illustrated as a density plot in gray. In the generic scenario, BMI was calculated using the generalized data of height and weight. MR: marketer risk; PR: prosecutor risk.

The age, height, weight, and BMI variables were converted from a numerical to a categorical scale during the anonymization process. In the use case–specific scenario, the degree of generalization in scale transformation was preconfigured to preserve relevant information. In the generic scenario, there were no restrictions in the generalization hierarchies. The resulting loss of information is visualized in Figure 6 for age and BMI for the subset of female participants who did not have diabetes. In the generic scenario, generalization of age leads to 20-year intervals at 50% PR+3.03% MR, 9.09% PR, and 3.03% PR. In contrast, as predefined, the intervals did not exceed 10 years in the use case–specific scenario. For BMI, generalization was more complex. In the generic scenario, BMI was calculated using the generalized data of height and weight (Figure S5 in Multimedia Appendix 1), which resulted in diverse, partly overlapping, intervals. As demonstrated in Figure S4 in Multimedia Appendix 1, the number of intervals decreased with increasing protection, while their length increased. This resulted in relevant information loss with intervals covering a range from normal weight (24.7 kg/cm^2^) to severe obesity (≥40 kg/cm^2^). As for age, the use case–specific scenario had restrictions in generalization hierarchies for BMI, which resulted in commonly accepted categories (normal weight, overweight, and obesity) and a good approximation to the original distribution. Reasonable semantics were maintained even at 3.03% PR. Thus, use case–specific configurations were important to obtain reasonable semantics of the variables. Height and weight were considered less relevant for the research focus and therefore removed in favor of preserving the BMI in the use case–specific scenario.

We additionally plotted information on the age distribution stratified by gender and the presence of diabetes mellitus for the original and the anonymized data at the selected privacy levels (Figure 4). In the generic scenario, the figure could only be replicated at 50% PR+9.09% MR due to the large intervals when stricter risk thresholds were enforced. By contrast, the use case–specific scenario maintained the original interval length.

## Discussion

### Principal Results

This study provides an in-depth view into the use of anonymization processes for sharing data from clinical studies. Based on a state-of-the-art threat modeling methodology and established risk models, a wide range of anonymization configurations was compared to study the privacy-utility trade-off. We further considered a use case–specific anonymization approach tailored toward our real-world application to optimize anonymization.

Our results exhibited quite high average privacy achieved for all records (<10% empirical average PR, ie, MR) even at the highest predefined thresholds for PR. At the same time, use case–specific utility at the data set–level was high (>90%) across all thresholds. Individual results disagreed in the sense of nonoverlapping 95% CIs, but this was rare and mainly occurred at 3.03% PR. We would not consider them as relevant in our descriptive analyses where no direct implications were drawn from individual estimates. General-purpose metrics, in contrast, underestimated the actual utility in our real-world application. The 95% CI overlap at the data set–level therefore seems to be a useful proxy for actual utility in descriptive analyses.

Based on our investigation, it is evident that use case–specific tailoring had a positive effect on the reproducibility. In assessing anthropometric data, for example, use case–specific tailoring unfolded its potential, BMI represents a screening tool for chronic disease and mortality, while weight and in particular height are not pathological factors by themselves [54]. Consequently, reasonable and use case–specific preprocessing (removal of weight and height) resulted in preserved utility, while generic configuration (removal of BMI) lost almost all the information.

### Comparison With Prior Work

While an increasing number of examples of real-world applications of anonymization algorithms are published [12,15,16], we did not come across any investigations that measured the reproducibility (eg, by 95% CI overlap) of descriptive real-world analyses except for prior work on the GCKD study. However, several studies focusing on preserving the utility of anonymized data for descriptive real-world analyses without explicitly introducing use case–specific measures have been published. For instance, in the Lean European Open Survey on patients infected with SARS-CoV-2, an anonymization pipeline using 9.09% PR as a threshold was established [25]. The anonymized data set was evaluated in selected clinical parameters with reported maximum frequency differences of only 0.11%. In addition, a real-world analysis on patients with stroke presented with low error rates [15]. Interestingly, the authors of this study evaluated a new method that limits the degree to which generalization is applied. Analogously to what we observed in the use case–specific scenario, these predefined settings resulted in anonymized data that are closer to the original data. In addition, prior work on anonymized data of the GCKD study demonstrated preserved descriptive characteristics at 2 selected privacy levels and highlighted the limitations of general-purpose utility metrics [28]. Similar to this study, the 95% CI overlap was used to confirm reproducibility, but it was not evaluated throughout an entire research project or across different anonymization processes, and only a small proportion of the risk-utility space was covered.

In inferential statistics in general, there is evidence for a lack of reproducibility. The evaluation of a low dimensionality data set—that contains only 2 variables that needed to be protected—concluded there were biased results across differently anonymized data [27]. The authors reported a decreasing accuracy of relative risk estimates by clustering analyses independent of the applied privacy model. Similarly, a use case–specific evaluation focusing on machine learning models for early acute kidney injury risk prediction identified a statistically relevant discrepancy in individual performance measures while at the same time preserving overall prediction accuracy [26]. This discrepancy points toward a need for multidimensional utility assessment. As stated earlier and shown in these published examples, individual estimate disagreements might or might not result in false implications depending on the affection of outcomes or potential confounders.

Data sharing has been mandated by several regulatory agencies [2] and is desirable for many reasons (eg, transparency, reproducibility, collaboration, and innovation). It is often subject to institutional policies and laws, such as the General Data Protection Regulation [5]. To promote data sharing, technical conditions to satisfy FAIR need to be realized. However, at the same time, privacy-enhancing technologies should be thoroughly assessed. In this context, utility concerns can pose a real threat and should be as much a part of discussion as privacy concerns. We want to encourage data sharing in a way that does not compromise patients’ privacy or research quality. The research communicated through our project can contribute to a better understanding of anonymization and potential pitfalls.

We also encourage regulators, policy makers, and society to openly discuss the costs—in terms of domain expert knowledge, time, technical requirements, and utility—that all stakeholders are willing to bear to maintain high levels of privacy. Our results highlight the weakness of generic anonymization when aiming to support disparate uses of the data when high levels of privacy need to be maintained. Conclusions might be either taking the extra costs of use case–specific tailoring and additional measures of control, agreeing on a lower privacy level in favor of high-quality research, or accepting the limitations of studies conducted on a generic anonymized data set.

### Limitations

While our evaluation included a comprehensive assessment, there are several limitations to this investigation. First, this study focused on measuring and reducing specific privacy concerns, namely, prosecutor and marketer reidentification risk, as well as a certain type of anonymization framework that uses generalization and suppression. There are certainly other privacy risks and anonymization algorithms, and it is possible that some may provide a better privacy-utility trade-off in certain scenarios. Second, we did not protect the data against sensitive attribute inference where confidential information is accessed indirectly through inference. We made this decision because we assumed a controlled access setting and the relevance of this risk as well as its possible countermeasures are controversial [55]. Third, the threat modeling approach we relied upon requires assumptions about the goals and possibilities of potential adversaries. Other researchers aiming to apply our technique could consider running a structured assessment among a panel of experts (eg, using the Delphi technique) to strengthen the reliability of the threat modeling. Finally, while descriptive analyses are a basic feature of almost any study, anonymization must also stand up to more complex statistics. Individual nonoverlapping 95% CIs as detected in this study might relevantly affect inferential statistics. In this context, the estimate agreement (ie, by 95% CI overlap) and direction of effect and statistical significance need to be considered [53].

### Conclusions

Against the background of increasing data sharing initiatives, it should be highlighted that utility concerns should be as much a part of discussion as privacy concerns. Our results highlight the weakness of generic anonymization when high levels of privacy are maintained. An anonymized data set aiming to support multiple disparate and possibly competing likely uses might allow exploratory analyses but may not be appropriate for drawing conclusions from individual analyses. This underscores the merit of applying case-specific tailoring and may justify its extra costs, for example, in terms of time and additional measures of control. However, the discussion about the acceptable costs, both financial and in terms of utility, that are required to uphold high levels of privacy should involve a broad spectrum of stakeholders. It should include domain experts, regulators, policy makers, patient representatives, and society at large.

## Appendix

Multimedia Appendix 1 Details on methods and additional results.

## Abbreviations

CKD: chronic kidney disease
eGFR: estimated glomerular filtration rate
FAIR: findable, accessible, interoperable, and reusable
GCKD: German Chronic Kidney Disease
MR: marketer risk
PR: prosecutor risk
UACR: urine albumin-to-creatinine ratio

## Data Availability

The data sets generated and analyzed during this study are not publicly available due to privacy risks but are available from the corresponding author on reasonable request.
