# Supplementary material for: The Costs of Anonymization: Case Study Using Clinical Data
Source: J Med Internet Res. 2024 Apr 24;26:e49445. doi: 10.2196/49445 (PMC11079766; doi:10.2196/49445)

**Multimedia Appendix 1. Details on methods and additional results.**

**Table S1.** Identification of variables with re-identification risks. To detect variables that could be used for re-identifying study participants, we adopted a two-step procedure. First, we used international guidelines to identify potentially linkable variables. We then performed semi-quantitative risk assessment of each variable transmitted by the study’s central coordination. We used the principles availability, replicability and distinguishability [40]. Each principle was quantified from low (1) to high (3). A score across all principles was calculated for each variable. We declared a score over 5 as criteria for the consideration as “risky” variable (in italic print). In availability, the quantification was based on phenotype and data access. We defined low risk as restricted access (e.g. laboratory values) and high risk as phenotypic features (e.g. gender). In replicability, low described a highly inconsistent variable over time (e.g. heart frequency) and high a mostly immutable variable (e.g. gender). In distinguishability, we estimated the variable’s prevalence in the reference group (German adults suffering from CKD) according to literature and expert knowledge. For low risk, the cut-off of prevalence was set over 50000 (e.g. use of antihypertensive medication); for high risk under 20000 (e.g. specific GFR estimates) according to recommendations [58]. BMI: body-mass index; SBP: systolic blood pressure; DBP: diastolic blood pressure; MAP: mean arterial pressure; eGFR: estimated glomerular filtration rate; MI: myocardial infarction; PTCA: percutaneous transluminal coronary angioplasty; PTA: percutaneous transluminal angioplasty.

| Variable | Scale | Semi-quantitative risk assessment | | | |
| --- | --- | --- | --- | --- | --- |
|  |  | Availability | Replicability | Distinguishability | Score |
|  |  |  |  |  |  |
| *Age (years)* | *Continuous* | *2* | *3* | *2* | *7* |
| *Gender* | *Nominal* | *3* | *3* | *1* | *7* |
| Family history - 1st degree relative with | | | | | |
| Stroke | Dichotomous | 1 | 2 | 1 | 4 |
| Myocardial infarction | Dichotomous | 1 | 2 | 1 | 4 |
| Hypertension | Dichotomous | 1 | 2 | 1 | 4 |
| Diabetes mellitus | Dichotomous | 1 | 2 | 1 | 4 |
| Kidney disease | Dichotomous | 1 | 2 | 1 | 4 |
| Renal stones | Dichotomous | 1 | 2 | 1 | 4 |
| Dialysis | Dichotomous | 1 | 2 | 1 | 4 |
| Kidney transplantation | Dichotomous | 1 | 2 | 2 | 5 |
| Smoking history | Dichotomous | 2 | 2 | 1 | 5 |
| Hospitalization in the past 5 years | Dichotomous | 1 | 2 | 1 | 4 |
| Anthropometric data | | | | | |
| *Height (cm)* | *Continuous* | *2* | *2* | *2* | *6* |
| *Weight (kg)* | *Continuous* | *2* | *2* | *3* | *7* |
| *BMI (kg/m2)* | *Continuous* | *2* | *2* | *2* | *6* |
| Pulse (bpm) | Continuous | 1 | 1 | 3 | 5 |
| SBP (mmHg) | Continuous | 1 | 1 | 3 | 5 |
| DBP (mmHg) | Continuous | 1 | 1 | 3 | 5 |
| MAP (mmHg) | Continuous | 1 | 1 | 3 | 5 |
| Kidney function measures | | | | | |
| Serum creatinine (mg/dl) | Continuous | 1 | 1 | 3 | 5 |
| Serum cystatin C (mg/l) | Continuous | 1 | 1 | 3 | 5 |
| eGFR (MDRD) (ml/min x 1.73 m2) | Continuous | 1 | 1 | 3 | 5 |
| eGFR (CKD-EPI Crea) (ml/min x 1.73 m2) | Continuous | 1 | 1 | 3 | 5 |
| eGFR (CKD-EPI Cys C) (ml/min x 1.73 m2) | Continuous | 1 | 1 | 3 | 5 |
| eGFR (CKD-EPI Crea Cys C) (ml/min x 1.73 m2) | Continuous | 1 | 1 | 3 | 5 |
| U-albumin/creatinine ratio (mg/g) | Continuous | 1 | 1 | 3 | 5 |
| Anti-hypertensives and diuretics | | | | | |
| ACE-inhibitors | Dichotomous | 1 | 2 | 1 | 4 |
| AII receptor blockers (ARB) | Dichotomous | 1 | 2 | 1 | 4 |
| ACE-inhibitors + ARB combined | Dichotomous | 1 | 2 | 1 | 4 |
| ACE-inhibitors or ARB | Dichotomous | 1 | 2 | 1 | 4 |
| Diuretics | Dichotomous | 1 | 2 | 1 | 4 |
| thiazides | Dichotomous | 1 | 2 | 1 | 4 |
| aldosterone antagonists | Dichotomous | 1 | 2 | 1 | 4 |
| loop diuretics | Dichotomous | 1 | 2 | 1 | 4 |
| Calcium channel blockers | Dichotomous | 1 | 2 | 1 | 4 |
| Beta blockers | Dichotomous | 1 | 2 | 1 | 4 |
| *Renal biopsy* | *Dichotomous* | *1* | *2* | *3* | *6* |
| Disease cause | | | | | |
| Diabetic nephropathy | Dichotomous | 2 | 2 | 1 | 5 |
| Vascular nephropathy | Dichotomous | 1 | 2 | 1 | 4 |
| Systemic disease | Dichotomous | 1 | 2 | 1 | 4 |
| Primary glomerulopathy | Dichotomous | 1 | 2 | 2 | 5 |
| Interstitial nephropathy | Dichotomous | 1 | 2 | 1 | 4 |
| Hereditary kidney disease | Dichotomous | 1 | 2 | 1 | 4 |
| Acute kidney injury | Dichotomous | 1 | 2 | 1 | 4 |
| Single kidney | Dichotomous | 1 | 2 | 2 | 5 |
| Obstructive nephropathy | Dichotomous | 1 | 2 | 1 | 4 |
| Miscallaneous | Dichotomous | 1 | 2 | 1 | 4 |
| Undetermined | Dichotomous | 1 | 2 | 1 | 4 |
| Leading cause of CKD | Nominal | 1 | 2 | 2 | 5 |
| Concomitant diseases | | | | | |
| Diabetes mellitus | Dichotomous | 1 | 2 | 1 | 4 |
| Arterial hypertension | Dichotomous | 1 | 2 | 1 | 4 |
| Cardiac valve replacement | Dichotomous | 1 | 2 | 1 | 4 |
| Coronary artery disease | Dichotomous | 1 | 2 | 1 | 4 |
| MI | Dichotomous | 1 | 2 | 1 | 4 |
| Bypass surgery | Dichotomous | 1 | 2 | 1 | 4 |
| PTCA | Dichotomous | 1 | 2 | 1 | 4 |
| Cerebrovascular disease | Dichotomous | 1 | 2 | 1 | 4 |
| Stroke | Dichotomous | 1 | 2 | 1 | 4 |
| Carotic surgery | Dichotomous | 1 | 2 | 1 | 4 |
| Carotic intervention | Dichotomous | 1 | 2 | 1 | 4 |
| Peripheral vascular disease | Dichotomous | 1 | 2 | 1 | 4 |
| Amputations | Dichotomous | 2 | 2 | 1 | 5 |
| Y-graft | Dichotomous | 1 | 2 | 1 | 4 |
| Other types of surgery | Dichotomous | 1 | 2 | 1 | 4 |
| PTA | Dichotomous | 2 | 2 | 1 | 5 |
| Cardiovascular disease | Dichotomous | 1 | 2 | 1 | 4 |
| Patient awareness period | Ordinal | 1 | 1 | 1 | 3 |
| Treatment period | Ordinal | 1 | 2 | 1 | 4 |
| Inclusion criteria | Nominal | 1 | 2 | 2 | 5 |
| Educational attainment | Nominal | 2 | 2 | 1 | 5 |

**Table S2.** Disease burden and risk profile of female participants who were nondiabetic within the GCKD cohort across anonymized datasets at moderate privacy levels (50% PR + 9.09% MR, 50% PR + 3.03% MR). Only parameters without scale transformation during the anonymization process are presented. Transformation and privacy models were applied as defined in the generic and Use case–specific scenario. Results are expressed as numbers (n) with percentages (%) referred to the numbers excluding missing data for categorical data and as mean or median with standard deviation (SD) or interquartile range (IQR) for numerical data. Proportion 95% confidence intervals (95% CI) were calculated based on the Wilson score interval and are displayed for the original data. PR: prosecutor risk; MR: marketer risk; bmp: beats per minute; (S)(D)BP: (systolic)(diastolic) blood pressure; eGFR: estimated glomerular filtration rate according to the MDRD equation; U: urine; UACR: urine albumin to creatinine ratio; ARB: AII receptor blockers.

|  |  |  | Original dataset | | | Generic scenario | | | | Use case–specific scenario | | | |
| --- | --- | --- | --- | --- | --- | --- | --- | --- | --- | --- | --- | --- | --- |
|  |  |  |  | | | 50% PR + 9.09% MR | | 50% PR + 3.03% MR | | 50% PR + 9.09% MR | | 50% PR + 3.03% MR | |
|  |  |  | n=1462 |  |  | n=1385 |  | n=1407 |  | n=1414 |  | n=1451 |  |
|  |  |  | n or mean | % or SD | 95% CI | n or mean | % or SD | n or mean | % or SD | n or mean | % or SD | n or mean | % or SD |
|  |  |  |  |  |  |  |  |  |  |  |  |  |  |
| Family history | | |  |  |  |  |  |  |  |  |  |  |  |
|  | Stroke | | 552 | 39.8 | 37.2-42.4 | 521 | 39.7 | 528 | 39.6 | 531 | 39.6 | 547 | 39.8 |
|  | Myocardial infarction | | 583 | 42.2 | 39.6-44.9 | 553 | 42.3 | 559 | 42.1 | 563 | 42.2 | 577 | 42.1 |
|  | Hypertension | | 1013 | 78.2 | 75.8-80.4 | 953 | 77.7 | 969 | 77.8 | 974 | 77.9 | 1004 | 78.1 |
|  | Diabetes | | 662 | 48.3 | 45.6-50.9 | 617 | 47.6 | 630 | 47.8 | 627 | 47.4 | 657 | 48.3 |
|  | Renal disease | | 444 | 32.9 | 30.4-35.5 | 418 | 32.7 | 426 | 32.8 | 429 | 32.9 | 440 | 32.9 |
|  | Renal stones | | 142 | 11.0 | 9.35-12.8 | 132 | 10.8 | 138 | 11.1 | 138 | 11.0 | 141 | 11.0 |
|  | Dialysis | | 110 | 8.2 | 6.84-9.86 | 100 | 7.9 | 106 | 8.3 | 105 | 8.1 | 108 | 8.1 |
|  | Kidney transplantation | | 49 | 3.7 | 2.74-4.84 | 41 | 3.2 | 45 | 3.5 | 45 | 3.5 | 47 | 3.5 |
| Smoking history | | |  |  |  |  |  |  |  |  |  |  |  |
|  | Current smokers | | 217 | 15.0 | 13.2-16.9 | 203 | 14.8 | 210 | 15.0 | 209 | 14.9 | 215 | 14.9 |
|  | Former smokers | | 427 | 29.4 | 27.1-31.9 | 403 | 29.3 | 408 | 29.2 | 415 | 29.6 | 425 | 29.5 |
|  | Never smokers | | 807 | 55.6 | 53.0-58.2 | 769 | 55.9 | 780 | 55.8 | 779 | 55.5 | 800 | 55.6 |
| Hospitalization past 5 years | | | 1011 | 70.6 | 68.1-72.9 | 954 | 70.3 | 969 | 70.3 | 936 | 954 | 971 | 70.1 |
| Pulse and blood pressure | | |  |  |  |  |  |  |  |  |  |  |  |
|  | Pulse (bpm) | | 71.3 | 11.5 | 70.7-71.9 | 71.1 | 11.3 | 71.1 | 11.3 | 71.2 | 11.4 | 71.2 | 11.5 |
|  | SBP (mmHg) | | 135.3 | 19.7 | 134.0-136.0 | 135.6 | 19.8 | 135.4 | 19.8 | 135.4 | 19.8 | 135.4 | 19.8 |
|  | DBP (mmHg) | | 80.6 | 11.0 | 80.1-81.2 | 80.6 | 11.0 | 80.5 | 11.0 | 80.5 | 11.0 | 80.6 | 11.0 |
|  | MAP (mmHg) | | 98.9 | 12.4 | 98.2-99.5 | 98.9 | 12.5 | 98.8 | 12.5 | 98.8 | 12.4 | 98.8 | 12.5 |
|  | BP < 130/80 mmHg | | 454 | 31.8 | 29.4-34.3 | 431 | 31.8 | 439 | 31.9 | 439 | 31.8 | 452 | 31.9 |
|  | BP < 140/90 mmHg | | 863 | 60.4 | 57.8-62.9 | 811 | 59.9 | 829 | 60.2 | 831 | 60.2 | 856 | 60.4 |
| Kidney function measures | | |  |  |  |  |  |  |  |  |  |  |  |
|  | Serum creatinine (mg/dl) | | 1.3 | 0.4 | 1.24-1.38 | 1.3 | 0.4 | 1.3 | 0.4 | 1.3 | 0.4 | 1.3 | 0.4 |
|  | Serum cystatin C (mg/l) | | 1.4 | 0.4 | 1.38-1.42 | 1.4 | 0.4 | 1.4 | 0.4 | 1.4 | 0.4 | 1.4 | 0.4 |
|  | eGFR (ml/min x 1.73 m^2^) | | 49.3 | 18.4 | 48.4-50.3 | 48.8 | 17.8 | 49.2 | 18.2 | 49.0 | 18.0 | 49.1 | 18.1 |
|  | eGFR >= 60 | | 292 | 20.2 | 18.1-22.3 | 261 | 19.0 | 279 | 20.0 | 271 | 19.3 | 286 | 19.9 |
|  | eGFR 45-59 | | 474 | 32.7 | 30.3-35.2 | 460 | 33.5 | 457 | 32.7 | 463 | 33.0 | 471 | 32.8 |
|  | eGFR 30-44 | | 544 | 37.5 | 35.1-40.1 | 523 | 38.0 | 528 | 37.8 | 535 | 38.2 | 544 | 37.8 |
|  | eGFR < 30 | | 139 | 9.6 | 8.15-11.3 | 131 | 9.5 | 132 | 9.5 | 132 | 9.4 | 137 | 9.5 |
|  | U-albumin/creatinine (mg/g), median; IQR | | 33.9 | 8.7-301.5 | 15.9-36.0 | 31.4 | 8.6-293.0 | 33.2 | 8.7-  293.1 | 32.4 | 8.6-293.0 | 33.2 | 8.7-  300.1 |
|  | UACR < 30 | | 696 | 48.4 | 45.8-51.0 | 670 | 49.1 | 673 | 48.6 | 680 | 48.9 | 694 | 48.6 |
|  | UACR 30-300 | | 382 | 26.5 | 24.3-28.9 | 359 | 26.3 | 372 | 26.8 | 370 | 26.6 | 377 | 26.4 |
|  | UACR > 300 | | 361 | 25.1 | 22.9-27.4 | 335 | 24.6 | 341 | 24.6 | 342 | 24.6 | 357 | 25.0 |
| Anti-hypertensives and diuretics | | | | | | | | | | | | | |
|  | ACE-inhibitors | | 553 | 37.8 | 35.3-40.4 | 525 | 37.9 | 535 | 38.0 | 539 | 38.1 | 548 | 37.8 |
|  | ARB | | 606 | 41.5 | 38.9-44.0 | 571 | 41.2 | 579 | 41.2 | 585 | 41.4 | 602 | 41.5 |
|  | ACE-inhibitors + ARB combined | | 70 | 4.8 | 3.78-6.04 | 67 | 4.8 | 68 | 4.8 | 67 | 4.7 | 69 | 4.8 |
|  | ACE-inhibitors or ARB | | 1019 | 69.7 | 67.3-72.0 | 962 | 69.5 | 978 | 69.5 | 990 | 70.0 | 1012 | 69.7 |
|  | Diuretics | | 659 | 45.1 | 42.5-47.7 | 622 | 44.9 | 634 | 45.1 | 641 | 45.3 | 656 | 45.2 |
|  |  | Thiazides | 307 | 21.0 | 19.0-23.2 | 291 | 21.0 | 297 | 21.1 | 296 | 20.9 | 305 | 21.0 |
|  |  | Aldosterone antagonists | 83 | 5.7 | 4.57-7.02 | 75 | 5.4 | 80 | 5.7 | 80 | 5.7 | 83 | 5.7 |
|  |  | Loop diuretics | 345 | 23.6 | 21.5-25.9 | 326 | 23.5 | 332 | 23.6 | 337 | 23.8 | 344 | 23.7 |
|  | Calcium channel blockers | | 391 | 26.7 | 24.5-29.1 | 374 | 27.0 | 377 | 26.8 | 381 | 26.9 | 388 | 26.7 |
|  | Beta blockers | | 640 | 43.8 | 41.2-46.4 | 613 | 44.3 | 617 | 43.9 | 627 | 44.3 | 639 | 44.0 |
| Renal biopsy | | | 468 | 32.0 | 29.7-34.5 | 427 | 30.8 | 442 | 31.4 | 445 | 31.5 | 462 | 31.8 |

**Table S3.** Disease burden and risk profile of female participants who were nondiabetic within the GCKD cohort across anonymized datasets at strict privacy levels (9.09% PR, 3.03% PR). Only parameters without scale transformation during the anonymization process are presented. Transformation and privacy models were applied as defined in the generic and Use case–specific scenario. Results are expressed as numbers (n) with percentages (%) referred to the numbers excluding missing data for categorical data and as mean or median with standard deviation (SD) or interquartile range (IQR) for numerical data. Proportion 95% confidence intervals (95% CI) were calculated based on the Wilson score interval and are displayed for the original data. ARB: AII receptor blocker; bmp: beats per minute; (S)(D)BP: (systolic)(diastolic) blood pressure; eGFR: estimated glomerular filtration rate according to the MDRD equation; PR: prosecutor risk; U: urine; UACR: urine albumin to creatinine ratio.

|  | Original dataset | | | | Generic scenario | | | | | | | | Use case–specific scenario | | | | | | |
| --- | --- | --- | --- | --- | --- | --- | --- | --- | --- | --- | --- | --- | --- | --- | --- | --- | --- | --- | --- |
|  |  | | | | 9.09% PR | | | | 3.03% PR | | | | 9.09% PR | | | 3.03% PR | | | |
|  | n=1462 |  | |  | n=1360 |  | | | n=1309 |  | | | n=1342 |  | | n=1218 |  | | |
|  | n or mean | % or SD | | 95% CI | n or mean | % or SD | | | n or mean | % or SD | | | n or mean | % or SD | | n or mean | % or SD | | |
|  |  |  | |  |  |  | | |  |  | | |  |  | |  |  | | |
| Family history |  |  | |  |  |  | | |  |  | | |  |  | |  |  | | |
| Stroke | 552 | 39.8 | 37.2-42.4 | | 510 | 39.5 | | | 495 | 40.0 | | | 499 | 39.3 | | 461 | 39.9 | |  |
| Myocardial infarction | 583 | 42.2 | 39.6-44.9 | | 539 | 42.0 | | | 518 | 41.9 | | | 536 | 42.3 | | 486 | 42.0 | |  |
| Hypertension | 1013 | 78.2 | 75.8-80.4 | | 938 | 77.8 | | | 903 | 78.1 | | | 923 | 77.7 | | 849 | 78.7 | |  |
| Diabetes | 662 | 48.3 | 45.6-50.9 | | 599 | 47.1 | | | 569 | 46.6 | | | 592 | 47.2 | | 529 | 46.5 | |  |
| Renal disease | 444 | 32.9 | 30.4-35.5 | | 412 | 32.8 | | | 398 | 33.1 | | | 407 | 33.0 | | 374 | 33.3 | |  |
| Renal stones | 142 | 11.0 | 9.35-12.8 | | 136 | 11.3 | | | 129 | 11.2 | | | 135 | 11.4 | | 126 | 11.7 | |  |
| Dialysis | 110 | 8.2 | 6.84-9.86 | | 100 | 8.0 | | | 93 | 7.8 | | | 97 | 7.9 | | 87 | 7.8 | |  |
| Kidney transplantation | 49 | 3.7 | 2.74-4.84 | | 43 | 3.4 | | | 41 | 3.4 | | | 40 | 3.3 | | 37 | 3.3 | |  |
| Smoking history |  |  | |  |  |  | | |  |  | | |  |  | |  |  | | |
| Current smokers | 217 | 15.0 | 13.2-16.9 | | 200 | 14.8 | | | 192 | 14.8 | | | 197 | 14.8 | | 174 | 14.4 | |  |
| Former smokers | 427 | 29.4 | 27.1-31.9 | | 395 | 29.2 | | | 385 | 29.6 | | | 397 | 29.8 | | 368 | 30.4 | |  |
| Never smokers | 807 | 55.6 | 53.0-58.2 | | 756 | 56.0 | | | 723 | 55.6 | | | 738 | 55.4 | | 667 | 55.2 | |  |
| Hospitalization past 5 years | 1011 | 70.6 | | 68.1-72.9 | 954 | 936 | | | 70.2 | 899 | | | 70.1 | 919 | | 69.9 | 830 | | |
| Pulse and blood pressure |  |  | |  |  |  | | |  |  | | |  |  | |  |  | | |
| Pulse (bpm) | 71.3 | 11.5 | 70.7-71.9 | | 71.1 | 11.3 | | | 70.9 | 11.3 | | | 71.1 | 11.2 | | 70.8 | 11.3 | |  |
| SBP (mmHg) | 135.3 | 19.7 | 134.0-136.0 | | 135.7 | 19.9 | | | 135.9 | 20.0 | | | 135.9 | 19.8 | | 136.4 | 20.0 | |  |
| DBP (mmHg) | 80.6 | 11.0 | 80.1-81.2 | | 80.6 | 11.1 | | | 80.5 | 11.1 | | | 80.5 | 10.9 | | 80.5 | 11.1 | |  |
| MAP (mmHg) | 98.9 | 12.4 | 98.2-99.5 | | 98.9 | 12.5 | | | 99.0 | 12.6 | | | 99.0 | 12.3 | | 99.1 | 12.5 | |  |
| BP < 130/80 mmHg | 454 | 31.8 | 29.4-34.3 | | 423 | 31.8 | | | 407 | 31.8 | | | 407 | 31.1 | | 361 | 30.4 | |  |
| BP < 140/90 mmHg | 863 | 60.4 | 57.8-62.9 | | 789 | 59.4 | | | 758 | 59.3 | | | 778 | 59.4 | | 692 | 58.2 | |  |
| Kidney function measures |  |  | |  |  |  | | |  |  | | |  |  | |  |  | | |
| Serum creatinine (mg/dl) | 1.3 | 0.4 | 1.24-1.28 | | 1.3 | 0.4 | | | 1.3 | 0.4 | | | 1.3 | 0.4 | | 1.3 | 0.4 | |  |
| Serum cystatin C (mg/l) | 1.4 | 0.4 | 1.38-1.42 | | 1.4 | 0.4 | | | 1.4 | 0.4 | | | 1.4 | 0.4 | | 1.4 | 0.4 | |  |
| eGFR (ml/min x 1.73 m^2^) | 49.3 | 18.4 | 48.4-50.3 | | 48.9 | 17.9 | | | 48.3 | 17.0 | | | 48.7 | 17.3 | | 47.9 | 16.3 | |  |
| eGFR >= 60 | 292 | 20.2 | 18.1-22.3 | | 259 | 19.2 | | | 242 | 18.6 | | | 252 | 18.9 | | 213 | 17.6 | |  |
| eGFR 45-59 | 474 | 32.7 | 30.3-35.2 | | 449 | 33.3 | | | 430 | 33.1 | | | 445 | 33.4 | | 405 | 33.5 | |  |
| eGFR 30-44 | 544 | 37.5 | 35.1-40.1 | | 512 | 38.0 | | | 504 | 38.8 | | | 512 | 38.4 | | 478 | 39.5 | |  |
| eGFR < 30 | 139 | 9.6 | 8.15-11.3 | | 129 | 9.6 | | | 124 | 9.5 | | | 123 | 9.2 | | 114 | 9.4 | |  |
| U-albumin/creatinine (mg/g), median; IQR | 33.9 | 8.7-301.5 | 15.9-36.0 | | 31.2 | 8.7-285.7 | | | 29.0 | 8.3-237.1 | | | 30.5 | 8.6-288.4 | | 26.1 | 8.0-  202.1 | |  |
| UACR < 30 | 696 | 48.4 | 45.8-51.0 | | 660 | 49.3 | | | 651 | 50.4 | | | 656 | 49.6 | | 631 | 52.5 | |  |
| UACR 30-300 | 382 | 26.5 | 24.3-28.9 | | 358 | 26.7 | | | 349 | 27.0 | | | 347 | 26.2 | | 314 | 26.1 | |  |
| UACR > 300 | 361 | 25.1 | 22.9-27.4 | | 321 | 24.0 | | | 292 | 22.6 | | | 320 | 24.2 | | 256 | 21.3 | |  |
| Anti-hypertensives and diuretics | | | | | | | | | | | | | | | | | | | |
| ACE-inhibitors | 553 | 37.8 | 35.3-40.4 | | 515 | | 37.9 | 485 | | | 37.8 | 507 | | | 37.8 | 446 | | 36.6 |  |
| ARB | 606 | 41.5 | 38.9-44.0 | | 563 | | 41.4 | 545 | | | 41.5 | 556 | | | 41.4 | 509 | | 41.8 |  |
| ACE-inhibitors + ARB combined | 70 | 4.8 | 3.78-6.04 | | 65 | 4.8 | | | 59 | 4.8 | | | 63 | 4.7 | | 50 | 4.1 | |  |
| ACE-inhibitors or ARB | 1019 | 69.7 | 67.3-72.0 | | 948 | 69.7 | | | 912 | 69.7 | | | 937 | 69.8 | | 855 | 70.2 | |  |
| Diuretics | 659 | 45.1 | 42.5-47.7 | | 615 | 45.2 | | | 607 | 45.2 | | | 612 | 45.6 | | 579 | 47.5 | |  |
| Thiazides | 307 | 21.0 | | 19.0-23.2 | 289 | 21.2 | | | 287 | 21.9 | | | 288 | 21.5 | | 276 | 22.7 | | |
| Aldosterone antagonists | 83 | 5.7 | | 4.6-7.0 | 75 | 5.5 | | | 72 | 5.5 | | | 76 | 5.7 | | 66 | 5.4 | | |
| Loop diuretics | 345 | 23.6 | | 21.5-25.9 | 321 | 23.6 | | | 315 | 24.1 | | | 320 | 23.8 | | 303 | 24.9 | | |
| Calcium channel blockers | 391 | 26.7 | 24.5-29.1 | | 368 | 27.1 | | | 359 | 26.7 | | | 363 | 27.0 | | 339 | 27.8 | |  |
| Beta blockers | 640 | 43.8 | 41.2-46.4 | | 598 | 44.0 | | | 592 | 44.0 | | | 597 | 44.5 | | 563 | 46.2 | |  |
| Renal biopsy | 468 | 32.0 | | 29.7-34.5 | 413 | 30.4 | | | 364 | 27.8 | | | 401 | 29.9 | | 299 | 24.5 | | |

**Table S4.** 95% CI overlap on estimate-level of the disease burden and risk profile of male participants and female participants who were diabetic across anonymized datasets at moderate privacy levels (50% PR + 9.09% MR, 50% PR + 3.03% MR). Only parameters without scale transformation during the anonymization process are presented. Transformation and privacy models were applied as defined in the generic and Use case–specific scenario. Proportion 95% confidence intervals (95% CI) based on the Wilson score interval served to calculate 95% CI length overlap compared to the original dataset as proposed by Karr et al. [24]. ARB: AII receptor blockers; (S)(D)BP: (systolic)(diastolic) blood pressure; eGFR: estimated glomerular filtration rate according to the MDRD equation; FD: female participants who were diabetic; MD: male participants who were diabetics; MND: male participants who were nondiabetic; MR: marketer risk; OC: overall cohort; bmp: beats per minute; PR: prosecutor risk; U: urine; UACR: urine albumin to creatinine ratio.

|  | | | Generic scenario | | | | | | | | Use case–specific scenario | | | | | | | |
| --- | --- | --- | --- | --- | --- | --- | --- | --- | --- | --- | --- | --- | --- | --- | --- | --- | --- | --- |
|  |  |  | 50% PR + 9.09% MR | | | | 50% PR + 3.03% MR | | | | 50% PR + 9.09% MR | | | | 50% PR + 3.03% MR | | | |
|  |  |  | FD | MND | MD | OC | FD | MND | MD | OC | FD | MND | MD | OC | FD | MND | MD | OC |
|  | | |  |  |  |  |  |  |  |  |  |  |  |  |  |  |  |  |
| Family history | | |  |  |  |  |  |  |  |  |  |  |  |  |  |  |  |  |
|  | Stroke | | 99.4 | 90.4 | 98.9 | 100.0 | 94.5 | 90.4 | 98.9 | 100.0 | 96.3 | 98.2 | 97.8 | 100.0 | 97.5 | 98.2 | 100.0 | 100.0 |
|  | Myocardial infarction | | 96.4 | 93.8 | 98.9 | 100.0 | 97.0 | 94.6 | 100.0 | 100.0 | 98.2 | 98.2 | 100.0 | 100.0 | 99.4 | 100.0 | 100.0 | 100.0 |
|  | Hypertension | | 97.9 | 97.3 | 96.8 | 100.0 | 93.8 | 100.0 | 98.9 | 100.0 | 94.4 | 100.0 | 100.0 | 100.0 | 98.6 | 100.0 | 100.0 | 100.0 |
|  | Diabetes | | 97.5 | 99.2 | 92.6 | 100.0 | 97.5 | 100.0 | 94.7 | 100.0 | 95.5 | 96.6 | 99.0 | 100.0 | 98.7 | 98.3 | 100.0 | 100.0 |
|  | Renal disease | | 98.1 | 98.1 | 97.7 | 100.0 | 98.8 | 98.1 | 98.8 | 100.0 | 94.9 | 97.2 | 94.1 | 100.0 | 98.7 | 99.1 | 96.5 | 100.0 |
|  | Renal stones | | 94.5 | 98.6 | 98.2 | 100.0 | 96.3 | 100.0 | 94.6 | 100.0 | 95.3 | 97.1 | 98.2 | 100.0 | 98.1 | 97.1 | 98.2 | 100.0 |
|  | Dialysis | | 97.5 | 100.0 | 98.1 | 100.0 | 86.1 | 100.0 | 100.0 | 100.0 | 97.5 | 96.4 | 96.0 | 100.0 | 100.0 | 100.0 | 96.0 | 100.0 |
|  | Kidney transplantation | | 94.7 | 95.0 | 96.2 | 100.0 | 92.0 | 95.0 | 96.2 | 100.0 | 97.5 | 100.0 | 100.0 | 100.0 | 100.0 | 100.0 | 96.2 | 100.0 |
| Smoking history | | |  |  |  |  |  |  |  |  |  |  |  |  |  |  |  |  |
|  | Current smokers | | 95.6 | 88.9 | 95.7 | 100.0 | 95.6 | 92.5 | 98.6 | 100.0 | 95.6 | 95.0 | 100.0 | 100.0 | 99.1 | 100.0 | 98.6 | 100.0 |
|  | Former smokers | | 99.3 | 91.1 | 89.2 | 100.0 | 99.3 | 96.4 | 92.3 | 100.0 | 97.9 | 96.4 | 90.1 | 100.0 | 98.6 | 98.2 | 96.7 | 100.0 |
|  | Never smokers | | 97.5 | 99.0 | 92.1 | 100.0 | 94.3 | 98.0 | 92.1 | 100.0 | 95.6 | 99.0 | 90.9 | 100.0 | 98.7 | 99.0 | 95.5 | 100.0 |
| Hospitalization past 5 years | | | 99.2 | 96.7 | 96.5 | 100.0 | 95.4 | 95.7 | 97.6 | 100.0 | 100.0 | 98.9 | 100.0 | 100.0 | 98.5 | 98.9 | 97.6 | 100.0 |
| Pulse and blood pressure | | |  |  |  |  |  |  |  |  |  |  |  |  |  |  |  |  |
|  | Pulse (bpm) | | 92.7 | 96.4 | 95.8 | 100.0 | 87.9 | 96.4 | 100.0 | 100.0 | 97.6 | 89.0 | 95.8 | 100.0 | 97.6 | 96.4 | 100.0 | 100.0 |
|  | SBP (mmHg) | | 87.5 | 100.0 | 100.0 | 100.0 | 100.0 | 100.0 | 100.0 | 100.0 | 100.0 | 100.0 | 75.0 | 100.0 | 100.0 | 100.0 | 100.0 | 100.0 |
|  | DBP (mmHg) | | 97.4 | 92.3 | 95.5 | 100.0 | 92.0 | 100.0 | 100.0 | 100.0 | 97.4 | 92.3 | 95.5 | 100.0 | 97.4 | 100.0 | 100.0 | 100.0 |
|  | MAP (mmHg) | | 95.2 | 100.0 | 75.0 | 100.0 | 100.0 | 100.0 | 100.0 | 100.0 | 97.6 | 96.7 | 75.0 | 100.0 | 97.6 | 100.0 | 100.0 | 100.0 |
|  | BP < 130/80 mmHg | | 93.5 | 93.7 | 93.7 | 100.0 | 99.4 | 90.7 | 94.9 | 100.0 | 98.7 | 97.9 | 93.7 | 100.0 | 100.0 | 100.0 | 98.7 | 100.0 |
|  | BP < 140/90 mmHg | | 97.6 | 98.2 | 96.8 | 100.0 | 99.4 | 98.2 | 97.8 | 100.0 | 98.8 | 97.4 | 94.6 | 100.0 | 99.4 | 99.1 | 97.8 | 100.0 |
| Kidney function measures | | |  |  |  |  |  |  |  |  |  |  |  |  |  |  |  |  |
|  | Serum creatinine (mg/dl) | | 100.0 | 100.0 | 100.0 | 100.0 | 100.0 | 100.0 | 100.0 | 100.0 | 100.0 | 100.0 | 100.0 | 100.0 | 100.0 | 100.0 | 100.0 | 100.0 |
|  | Serum cystatin C (mg/l) | | 100.0 | 100.0 | 100.0 | 100.0 | 100.0 | 100.0 | 100.0 | 100.0 | 100.0 | 100.0 | 100.0 | 100.0 | 100.0 | 100.0 | 100.0 | 100.0 |
|  | eGFR (MDRD) (ml/min x 1.73 m2) | | 81.7 | 88.2 | 86.7 | 100.0 | 76.0 | 94.1 | 93.3 | 100.0 | 89.8 | 97.1 | 89.8 | 100.0 | 92.0 | 97.1 | 93.3 | 100.0 |
|  | eGFR >= 60 | | 90.4 | 84.2 | 94.1 | 100.0 | 82.7 | 89.5 | 100.0 | 100.0 | 94.2 | 96.0 | 89.6 | 100.0 | 94.2 | 97.4 | 95.5 | 100.0 |
|  | eGFR 45-59 | | 95.9 | 91.8 | 96.6 | 100.0 | 96.6 | 90.7 | 95.5 | 100.0 | 97.3 | 96.3 | 93.2 | 100.0 | 98.6 | 98.1 | 97.7 | 100.0 |
|  | eGFR 30-44 | | 96.9 | 95.6 | 96.7 | 100.0 | 94.5 | 99.1 | 97.8 | 100.0 | 100.0 | 99.1 | 100.0 | 100.0 | 99.4 | 98.2 | 97.8 | 100.0 |
|  | eGFR < 30 | | 91.7 | 96.0 | 96.4 | 100.0 | 90.0 | 94.6 | 96.4 | 100.0 | 98.3 | 100.0 | 100.0 | 100.0 | 98.3 | 100.0 | 96.4 | 100.0 |
|  | U-albumin/creatinine (mg/g), median; IQR | | 83.0 | 89.1 | 96.2 | 100.0 | 91.1 | 99.6 | 97.2 | 100.0 | 89.0 | 94.7 | 97.8 | 100.0 | 93.2 | 96.1 | 97.8 | 100.0 |
|  | UACR < 30 | | 97.5 | 95.5 | 84.5 | 100.0 | 97.5 | 98.2 | 94.4 | 100.0 | 94.4 | 99.1 | 89.9 | 100.0 | 96.9 | 99.1 | 95.5 | 100.0 |
|  | UACR 30-300 | | 98.6 | 97.2 | 97.7 | 100.0 | 97.2 | 93.3 | 96.6 | 100.0 | 95.1 | 97.2 | 97.7 | 100.0 | 95.1 | 100.0 | 97.7 | 100.0 |
|  | UACR > 300 | | 99.2 | 92.6 | 82.4 | 100.0 | 94.4 | 94.4 | 94.1 | 100.0 | 98.4 | 96.3 | 94.1 | 100.0 | 99.2 | 99.1 | 94.1 | 100.0 |
| Anti-hypertensives and diuretics | | |  |  |  |  |  |  |  |  |  |  |  |  |  |  |  |  |
|  | ACE-inhibitors | | 95.7 | 99.1 | 94.6 | 100.0 | 98.1 | 99.1 | 97.8 | 100.0 | 100.0 | 99.1 | 100.0 | 100.0 | 98.8 | 98.2 | 95.7 | 100.0 |
|  | ARB | | 96.3 | 100.0 | 97.9 | 100.0 | 98.8 | 98.2 | 96.7 | 100.0 | 99.4 | 98.2 | 98.9 | 100.0 | 99.4 | 99.1 | 95.6 | 100.0 |
|  | ACE-inhibitors + ARB combined | | 85.3 | 97.2 | 92.9 | 100.0 | 88.0 | 100.0 | 98.3 | 100.0 | 94.7 | 95.8 | 100.0 | 100.0 | 97.4 | 98.6 | 100.0 | 100.0 |
|  | ACE-inhibitors or ARB | | 81.5 | 98.9 | 96.1 | 100.0 | 90.1 | 98.9 | 96.1 | 100.0 | 93.1 | 93.3 | 97.5 | 100.0 | 96.9 | 98.9 | 98.7 | 100.0 |
|  | Diuretics | | 96.4 | 94.5 | 98.9 | 100.0 | 94.1 | 95.6 | 95.7 | 100.0 | 99.3 | 100.0 | 97.8 | 100.0 | 100.0 | 100.0 | 95.7 | 100.0 |
|  |  | Thiazides | 95.2 | 93.3 | 95.1 | 100.0 | 95.2 | 96.3 | 98.7 | 100.0 | 99.3 | 98.1 | 96.2 | 100.0 | 100.0 | 100.0 | 98.7 | 100.0 |
|  |  | Aldosterone antagonists | 87.1 | 98.6 | 89.4 | 100.0 | 89.1 | 94.4 | 95.7 | 100.0 | 98.0 | 97.2 | 95.7 | 100.0 | 98.0 | 98.6 | 97.8 | 100.0 |
|  |  | Loop diuretics | 95.7 | 98.2 | 94.1 | 100.0 | 93.2 | 96.4 | 92.9 | 100.0 | 100.0 | 98.2 | 94.1 | 100.0 | 99.4 | 99.1 | 96.5 | 100.0 |
|  | Calcium channel blockers | | 92.0 | 99.1 | 94.5 | 100.0 | 96.3 | 99.1 | 100.0 | 100.0 | 98.1 | 98.2 | 97.8 | 100.0 | 99.4 | 97.4 | 98.9 | 100.0 |
|  | Beta blockers | | 92.2 | 96.2 | 96.8 | 100.0 | 90.2 | 95.2 | 93.5 | 100.0 | 97.4 | 95.2 | 93.5 | 100.0 | 96.1 | 98.0 | 96.7 | 100.0 |
| Renal biopsy | | | 75.0 | 88.3 | 81.8 | 61.3 | 71.2 | 94.9 | 90.9 | 77.6 | 82.7 | 92.3 | 88.6 | 77.6 | 92.3 | 100.0 | 95.5 | 92.0 |

**Table S5.** 95% CI overlap on estimate-level of the disease burden and risk profile of male participants and female participants who were diabetic across anonymized datasets across anonymized datasets at strict privacy levels (9.09% PR, 3.03% PR). Only parameters without scale transformation during the anonymization process are presented. Transformation and privacy models were applied as defined in the generic and Use case–specific scenario. Proportion 95% confidence intervals (95% CI) based on the Wilson score interval served to calculate 95% CI length overlap compared to the original dataset as proposed by Karr et al. [24]. ARB: AII receptor blockers; (S)(D)BP: (systolic)(diastolic) blood pressure; eGFR: estimated glomerular filtration rate according to the MDRD equation; FD: female participants who were diabetic; MD: male participants who were diabetics; MND: male participants who were nondiabetic; MR: marketer risk; OC: overall cohort; bmp: beats per minute; PR: prosecutor risk; U: urine; UACR: urine albumin to creatinine ratio.

|  | | | Generic scenario | | | | | | | | Use case–specific scenario | | | | | | | |
| --- | --- | --- | --- | --- | --- | --- | --- | --- | --- | --- | --- | --- | --- | --- | --- | --- | --- | --- |
|  |  |  | 9.09% PR | | | | 3.03% PR | | | | 9.09% PR | | | | 3.03% PR | | | |
|  |  |  | FD | MND | MD | OC | FD | MND | MD | OC | FD | MND | MD | OC | FD | MND | MD | OC |
|  | | |  |  |  |  |  |  |  |  |  |  |  |  |  |  |  |  |
| Family history | | |  |  |  |  |  |  |  |  |  |  |  |  |  |  |  |  |
|  | Stroke | | 97.0 | 93.9 | 92.3 | 100.0 | 98.2 | 92.2 | 91.3 | 100.0 | 96.4 | 95.7 | 100.0 | 100.0 | 95.8 | 96.5 | 94.5 | 100.0 |
|  | Myocardial infarction | | 98.2 | 95.6 | 96.8 | 100.0 | 96.4 | 94.6 | 89.4 | 100.0 | 98.8 | 98.2 | 98.9 | 100.0 | 98.2 | 93.8 | 92.5 | 100.0 |
|  | Hypertension | | 93.2 | 95.5 | 94.6 | 100.0 | 99.3 | 93.7 | 91.5 | 100.0 | 97.9 | 99.1 | 96.8 | 100.0 | 95.2 | 93.7 | 97.9 | 100.0 |
|  | Diabetes | | 97.6 | 95.7 | 98.0 | 100.0 | 95.6 | 94.0 | 92.9 | 100.0 | 92.4 | 98.3 | 90.5 | 100.0 | 93.1 | 93.1 | 84.6 | 100.0 |
|  | Renal disease | | 98.2 | 95.4 | 97.7 | 100.0 | 98.1 | 94.4 | 96.7 | 100.0 | 93.8 | 98.1 | 94.1 | 100.0 | 95.7 | 96.3 | 92.1 | 100.0 |
|  | Renal stones | | 98.2 | 98.6 | 96.6 | 100.0 | 92.6 | 95.7 | 96.6 | 100.0 | 94.5 | 100.0 | 96.6 | 100.0 | 91.8 | 98.6 | 91.5 | 100.0 |
|  | Dialysis | | 87.5 | 91.3 | 96.0 | 100.0 | 87.2 | 98.3 | 96.3 | 100.0 | 92.5 | 98.3 | 98.1 | 100.0 | 96.3 | 98.3 | 96.3 | 100.0 |
|  | Kidney transplantation | | 89.2 | 95.0 | 96.2 | 100.0 | 94.7 | 95.0 | 83.3 | 100.0 | 94.7 | 95.0 | 96.2 | 100.0 | 92.4 | 95.0 | 88.1 | 100.0 |
| Smoking history | | |  |  |  |  |  |  |  |  |  |  |  |  |  |  |  |  |
|  | Current smokers | | 93.0 | 98.8 | 97.2 | 100.0 | 92.9 | 93.8 | 60.9 | 100.0 | 99.1 | 91.4 | 95.7 | 100.0 | 85.0 | 100.0 | 78.3 | 100.0 |
|  | Former smokers | | 95.3 | 95.6 | 92.6 | 100.0 | 98.6 | 90.3 | 53.3 | 100.0 | 97.3 | 90.3 | 91.3 | 100.0 | 88.5 | 96.4 | 60.3 | 100.0 |
|  | Never smokers | | 89.5 | 96.0 | 92.1 | 100.0 | 94.3 | 95.0 | 83.6 | 100.0 | 97.5 | 97.0 | 95.5 | 100.0 | 98.8 | 97.0 | 77.8 | 100.0 |
| Hospitalization past 5 years | | | 98.5 | 97.9 | 100.0 | 100.0 | 97.7 | 93.5 | 88.4 | 100.0 | 99.2 | 95.7 | 97.6 | 100.0 | 98.5 | 97.9 | 98.8 | 100.0 |
| Pulse and blood pressure | | |  |  |  |  |  |  |  |  |  |  |  |  |  |  |  |  |
|  | Pulse (bpm) | | 83.0 | 96.4 | 87.1 | 100.0 | 87.9 | 96.4 | 81.8 | 100.0 | 95.5 | 96.4 | 87.1 | 100.0 | 92.7 | 96.4 | 78.4 | 100.0 |
|  | SBP (mmHg) | | 100.0 | 100.0 | 75.0 | 100.0 | 100.0 | 100.0 | 50.0 | 100.0 | 100.0 | 100.0 | 100.0 | 100.0 | 87.5 | 100.0 | 50.0 | 100.0 |
|  | DBP (mmHg) | | 84.2 | 89.0 | 95.5 | 100.0 | 84.2 | 96.4 | 95.5 | 100.0 | 94.7 | 89.0 | 95.5 | 100.0 | 89.5 | 92.3 | 85.9 | 100.0 |
|  | MAP (mmHg) | | 95.2 | 93.3 | 75.0 | 100.0 | 90.5 | 100.0 | 0.0 | 100.0 | 97.6 | 93.3 | 100.0 | 100.0 | 100.0 | 93.3 | 75.0 | 100.0 |
|  | BP < 130/80 mmHg | | 98.1 | 88.7 | 95.1 | 100.0 | 98.0 | 96.9 | 82.6 | 100.0 | 93.5 | 95.8 | 96.2 | 100.0 | 90.9 | 92.8 | 90.1 | 100.0 |
|  | BP < 140/90 mmHg | | 98.2 | 98.2 | 97.9 | 100.0 | 96.9 | 97.4 | 75.9 | 100.0 | 98.8 | 100.0 | 98.9 | 100.0 | 93.4 | 91.2 | 85.1 | 100.0 |
| Kidney function measures | | |  |  |  |  |  |  |  |  |  |  |  |  |  |  |  |  |
|  | Serum creatinine (mg/dl) | | 100.0 | 100.0 | 100.0 | 100.0 | 100.0 | 100.0 | 100.0 | 100.0 | 100.0 | 100.0 | 100.0 | 100.0 | 100.0 | 100.0 | 100.0 | 100.0 |
|  | Serum cystatin C (mg/l) | | 100.0 | 100.0 | 100.0 | 100.0 | 100.0 | 100.0 | 100.0 | 100.0 | 100.0 | 100.0 | 100.0 | 100.0 | 100.0 | 100.0 | 100.0 | 100.0 |
|  | eGFR (MDRD) (ml/min x 1.73 m2) | | 72.0 | 94.1 | 86.7 | 100.0 | 85.7 | 100.0 | 53.3 | 100.0 | 80.0 | 88.2 | 86.7 | 100.0 | 85.7 | 94.1 | 34.5 | 100.0 |
|  | eGFR >= 60 | | 88.7 | 89.5 | 91.2 | 100.0 | 83.8 | 92.1 | 58.8 | 100.0 | 91.4 | 86.8 | 82.4 | 100.0 | 87.6 | 93.5 | 50.0 | 100.0 |
|  | eGFR 45-59 | | 87.9 | 95.4 | 97.7 | 100.0 | 95.2 | 88.1 | 82.3 | 100.0 | 91.2 | 95.4 | 84.1 | 100.0 | 93.3 | 95.4 | 82.3 | 100.0 |
|  | eGFR 30-44 | | 93.9 | 99.1 | 94.5 | 100.0 | 93.3 | 99.1 | 84.0 | 100.0 | 88.3 | 99.1 | 94.5 | 100.0 | 92.7 | 99.1 | 76.1 | 100.0 |
|  | eGFR < 30 | | 82.1 | 97.3 | 96.4 | 100.0 | 99.2 | 89.2 | 93.2 | 100.0 | 97.5 | 93.3 | 85.7 | 100.0 | 93.4 | 100.0 | 91.3 | 100.0 |
|  | U-albumin/creatinine (mg/g), median; IQR | | 83.0 | 94.2 | 95.5 | 100.0 | 63.9 | 71.2 | 99.0 | 100.0 | 77.9 | 83.8 | 94.6 | 100.0 | 19.8 | 59.0 | 96.6 | 100.0 |
|  | UACR < 30 | | 98.2 | 98.2 | 91.2 | 100.0 | 88.9 | 98.2 | 74.8 | 100.0 | 94.5 | 95.5 | 80.0 | 100.0 | 78.6 | 95.5 | 63.8 | 100.0 |
|  | UACR 30-300 | | 98.0 | 96.3 | 96.6 | 100.0 | 98.6 | 91.4 | 88.7 | 100.0 | 93.1 | 95.2 | 93.0 | 100.0 | 93.8 | 99.1 | 84.1 | 100.0 |
|  | UACR > 300 | | 96.8 | 98.1 | 88.4 | 100.0 | 83.9 | 92.6 | 62.8 | 100.0 | 99.2 | 90.7 | 73.0 | 100.0 | 79.0 | 94.4 | 47.1 | 100.0 |
| Anti-hypertensives and diuretics | | |  |  |  |  |  |  |  |  |  |  |  |  |  |  |  |  |
|  | ACE-inhibitors | | 92.1 | 94.8 | 98.9 | 100.0 | 99.4 | 99.1 | 96.9 | 100.0 | 96.9 | 97.4 | 94.6 | 100.0 | 94.5 | 93.8 | 83.0 | 100.0 |
|  | ARB | | 96.4 | 95.7 | 97.9 | 100.0 | 98.2 | 85.2 | 96.9 | 100.0 | 96.9 | 96.5 | 96.7 | 100.0 | 97.6 | 89.5 | 88.3 | 100.0 |
|  | ACE-inhibitors + ARB combined | | 75.7 | 98.6 | 98.3 | 100.0 | 89.5 | 93.2 | 91.3 | 100.0 | 81.1 | 97.2 | 96.4 | 100.0 | 80.0 | 100.0 | 93.2 | 100.0 |
|  | ACE-inhibitors or ARB | | 86.4 | 98.9 | 97.5 | 100.0 | 88.6 | 91.3 | 91.3 | 100.0 | 85.5 | 96.7 | 97.5 | 100.0 | 84.9 | 94.5 | 97.5 | 100.0 |
|  | Diuretics | | 97.1 | 95.7 | 100.0 | 100.0 | 88.2 | 98.9 | 66.0 | 100.0 | 94.9 | 98.9 | 98.9 | 100.0 | 84.7 | 97.8 | 70.2 | 100.0 |
|  |  | Thiazides | 94.6 | 96.3 | 92.6 | 100.0 | 99.3 | 90.6 | 76.6 | 100.0 | 96.0 | 97.2 | 96.2 | 100.0 | 98.7 | 99.1 | 71.7 | 100.0 |
|  |  | Aldosterone antagonists | 88.2 | 90.4 | 93.7 | 100.0 | 96.1 | 83.3 | 85.1 | 100.0 | 92.2 | 95.9 | 100.0 | 100.0 | 97.1 | 97.2 | 87.7 | 100.0 |
|  |  | Loop diuretics | 97.0 | 97.4 | 89.4 | 100.0 | 86.2 | 99.1 | 80.6 | 100.0 | 99.4 | 96.4 | 98.8 | 100.0 | 86.4 | 96.4 | 87.5 | 100.0 |
|  | Calcium channel blockers | | 92.0 | 93.8 | 94.5 | 100.0 | 95.0 | 91.3 | 77.5 | 100.0 | 95.0 | 95.6 | 95.6 | 100.0 | 91.4 | 97.4 | 69.6 | 100.0 |
|  | Beta blockers | | 77.9 | 98.1 | 94.6 | 100.0 | 87.6 | 94.3 | 44.7 | 100.0 | 92.2 | 95.2 | 86.0 | 100.0 | 88.3 | 97.1 | 44.7 | 100.0 |
| Renal biopsy | | | 65.4 | 84.6 | 77.3 | 52.0 | 55.0 | 98.8 | 62.9 | 4.1 | 38.1 | 80.5 | 79.5 | 36.7 | 0.0 | 77.9 | 33.7 | 0.0 |

**Table S6.** 95% CI overlap of characteristics stratified by inclusion criteria on estimate-level across anonymized datasets. Only parameters without scale transformation during the anonymization process are presented. Transformation and privacy models were applied as defined in the generic and Use case–specific scenario. Proportion 95% confidence intervals (95% CI) based on the Wilson score interval served to calculate 95% CI length overlap compared to the original dataset as proposed by Karr et al [24]. Non-affected variables (100% 95% CI overlap) are not shown. The main inclusion criteria for renal function at screening were used as stratification criteria (eGFR 30-60ml/min x 1.73 m2 (GFR) and overt proteinuria at eGFR > 60ml/min x 1.73 m2 (OVR)). eGFR: estimated glomerular filtration rate according to the MDRD equation; MR: marketer risk; PR: prosecutor risk.

|  | Generic scenario | | | | | | | | Use case–specific scenario | | | | | | | |
| --- | --- | --- | --- | --- | --- | --- | --- | --- | --- | --- | --- | --- | --- | --- | --- | --- |
|  | 50% PR + 9.09% MR | | 50% PR + 3.03% MR | | 9.09% PR | | 3.03% PR | | 50% PR + 9.09% MR | | 50% PR + 3.03% MR | | 9.09% PR | | 3.03% PR | |
|  | GFR | OVR | GFR | OVR | GFR | OVR | GFR | OVR | GFR | OVR | GFR | OVR | GFR | OVR | GFR | OVR |
|  |  |  |  |  |  |  |  |  |  |  |  |  |  |  |  |  |
| Male gender | 94.8 | 83.0 | 92.9 | 88.4 | 89.3 | 84.0 | 98.3 | 83.7 | 92.9 | 86.9 | 100.0 | 97.4 | 71.4 | 72.2 | 45.6 | 42.8 |
| Renal biopsy | 66.7 | 97.4 | 79.2 | 96.3 | 54.2 | 95.9 | 20.4 | 86.2 | 83.3 | 94.8 | 95.8 | 99.5 | 45.8 | 94.4 | 0.0 | 52.9 |

**Table S7.** 95% CI overlap of biopsy rate per leading cause on estimate-level across anonymized datasets. Transformation and privacy models were applied as defined in the generic and Use case–specific scenario. Proportion 95% confidence intervals (95% CI) based on the Wilson score interval served to calculate 95% CI length overlap compared to the original dataset as proposed by Karr et al [24]. Only biopsy rate was affected in this table. All other variables (not shown) have 100% 95% CI overlap. MR: marketer risk; PR: prosecutor risk.

|  |  | Generic scenario | | | | Use case–specific scenario | | | |
| --- | --- | --- | --- | --- | --- | --- | --- | --- | --- |
|  |  | 50% PR + 9.09% MR | 50% PR + 3.03% MR | 9.09% PR | 3.03% PR | 50% PR + 9.09% MR | 50% PR + 3.03% MR | 9.09% PR | 3.03% PR |
|  | |  |  |  |  |  |  |  |  |
| Leading cause of CKD | |  |  |  |  |  |  |  |  |
|  | Diabetic nephropathy | 91.6 | 94.9 | 89.7 | 88.2 | 87.7 | 93.1 | 80.7 | 80.7 |
|  | Vascular nephropathy | 96.0 | 93.2 | 98.4 | 95.7 | 95.6 | 99.2 | 92.7 | 80.7 |
|  | Systemic disease | 100.0 | 100.0 | 83.9 | 90.3 | 95.1 | 100.0 | 82.0 | 76.8 |
|  | Primary glomerulopathy | 82.0 | 90.3 | 90.3 | 68.9 | 95.1 | 100.0 | 85.3 | 63.4 |
|  | Interstitial nephropathy | 90.4 | 94.8 | 81.5 | 72.1 | 98.3 | 98.2 | 84.9 | 50.5 |
|  | Acute kidney injury | 93.1 | 88.4 | 89.8 | 78.9 | 90.7 | 96.5 | 92.1 | 65.1 |
|  | Single kidney | 82.3 | 91.0 | 93.5 | 78.8 | 92.5 | 100.0 | 80.2 | 52.2 |
|  | Hereditary kidney disease | 94.6 | 85.2 | 85.2 | 87.5 | 94.6 | 92.4 | 89.8 | 89.8 |
|  | Obstructive nephropathy | 96.2 | 96.7 | 95.7 | 93.6 | 97.8 | 98.9 | 95.7 | 80.7 |
|  | Miscellaneous | 96.4 | 92.6 | 90.8 | 85.4 | 98.8 | 98.2 | 93.5 | 78.3 |
|  | Undetermined | 97.1 | 96.6 | 96.5 | 97.8 | 96.6 | 100.0 | 94.9 | 91.9 |

**Table S8.** 95% CI overlap of cardiovascular disease burden on estimate-level across anonymized datasets at moderate privacy levels (50% PR + 9.09% MR, 50% PR + 3.03% MR). Only parameters without scale transformation during the anonymization process are presented. Transformation and privacy models were applied as defined in the generic and Use case–specific scenario. Proportion 95% confidence intervals (95% CI) based on the Wilson score interval served to calculate 95% CI length overlap compared to the original dataset as proposed by Karr et al. [24]. Only affected variables are displayed. All estimates of the overall cohort were not affected (100% 95% CI overlap) and are not listed in the table. FD: female participants who were diabetic; FND: female participants who were non diabetic; MD: male participants who were diabetic; MI: myocardial infarction; MND: male participants who were non diabetic; MR: marketer risk; PR: prosecutor risk; PTA: percutaneous transluminal angioplasty; PTCA: percutaneous transluminal coronary angioplasty.

|  |  | Generic scenario | | | | | | | | Use case–specific scenario | | | | | | | |
| --- | --- | --- | --- | --- | --- | --- | --- | --- | --- | --- | --- | --- | --- | --- | --- | --- | --- |
|  |  | 50% PR + 9.09% MR | | | | 50% PR + 3.03% MR | | | | 50% PR + 9.09% MR | | | | 50% PR + 3.03% MR | | | |
|  |  | FND | FD | MND | MD | FND | FD | MND | MD | FND | FD | MND | MD | FND | FD | MND | MD |
|  | |  |  |  |  |  |  |  |  |  |  |  |  |  |  |  |  |
| Cardiovascular disease | | 85.4 | 88.1 | 93.0 | 85.1 | 88.9 | 93.7 | 94.7 | 89.7 | 92.5 | 98.1 | 94.7 | 93.0 | 96.3 | 98.1 | 100.0 | 100.0 |
| Arterial hypertension | | 100.0 | 100.0 | 95.0 | 93.8 | 94.9 | 100.0 | 95.0 | 81.3 | 91.6 | 95.8 | 100.0 | 97.1 | 100.0 | 95.8 | 100.0 | 100.0 |
| Cardiac valve replacement | | 96.2 | 94.8 | 97.4 | 96.4 | 96.2 | 94.8 | 97.4 | 100.0 | 96.2 | 98.3 | 100.0 | 100.0 | 100.0 | 98.3 | 100.0 | 100.0 |
| Coronary artery disease | | 91.8 | 90.6 | 93.6 | 83.3 | 91.8 | 90.6 | 95.4 | 88.9 | 90.0 | 99.3 | 94.4 | 91.7 | 96.7 | 99.3 | 98.1 | 95.8 |
|  | MI | 97.7 | 90.4 | 93.7 | 86.2 | 97.7 | 91.5 | 92.5 | 93.1 | 95.2 | 99.0 | 95.7 | 94.8 | 100.0 | 99.0 | 98.9 | 98.3 |
|  | Bypass surgery | 95.8 | 93.8 | 93.7 | 91.5 | 95.8 | 93.8 | 96.2 | 93.4 | 95.8 | 97.5 | 96.2 | 93.4 | 100.0 | 100.0 | 98.7 | 97.8 |
|  | PTCA | 89.0 | 94.1 | 92.0 | 88.9 | 92.7 | 93.4 | 98.0 | 93.5 | 90.6 | 98.3 | 96.0 | 93.5 | 98.1 | 99.2 | 99.0 | 96.8 |
| Cerebrovascular disease | | 86.8 | 98.1 | 96.3 | 92.9 | 90.6 | 96.1 | 96.3 | 92.9 | 96.2 | 97.0 | 96.3 | 96.4 | 96.2 | 100.0 | 97.5 | 100.0 |
|  | Stroke | 85.8 | 99.0 | 96.0 | 94.2 | 93.9 | 95.9 | 96.0 | 92.0 | 93.9 | 96.9 | 97.3 | 96.0 | 98.0 | 100.0 | 100.0 | 100.0 |
|  | Carotic surgery | 96.2 | 96.2 | 100.0 | 100.0 | 96.2 | 96.2 | 100.0 | 100.0 | 96.2 | 98.0 | 97.6 | 96.9 | 100.0 | 100.0 | 100.0 | 100.0 |
|  | Carotic intervention | 100.0 | 96.2 | 100.0 | 100.0 | 90.0 | 96.2 | 100.0 | 96.2 | 95.0 | 100.0 | 100.0 | 96.2 | 95.0 | 100.0 | 100.0 | 100.0 |
| Peripheral vascular disease | | 93.9 | 94.2 | 96.6 | 96.2 | 95.8 | 99.0 | 100.0 | 98.0 | 100.0 | 99.0 | 100.0 | 98.0 | 100.0 | 98.0 | 96.5 | 100.0 |
|  | Amputations | 96.7 | 94.4 | 98.4 | 93.3 | 96.7 | 98.1 | 100.0 | 96.7 | 96.7 | 96.2 | 96.7 | 100.0 | 100.0 | 100.0 | 93.3 | 100.0 |
|  | Y-graft | 94.4 | 96.2 | 96.9 | 100.0 | 94.4 | 96.2 | 96.9 | 100.0 | 94.4 | 100.0 | 100.0 | 100.0 | 100.0 | 100.0 | 100.0 | 100.0 |
|  | Other types of surgery | 96.9 | 94.9 | 100.0 | 100.0 | 94.1 | 94.9 | 97.7 | 100.0 | 96.9 | 98.3 | 100.0 | 100.0 | 96.9 | 100.0 | 97.7 | 100.0 |
|  | PTA | 96.9 | 97.6 | 98.5 | 100.0 | 100.0 | 97.6 | 95.4 | 97.2 | 100.0 | 97.6 | 100.0 | 97.2 | 100.0 | 100.0 | 100.0 | 97.2 |

**Table S9.** 95% CI overlap of cardiovascular disease burden on estimate-level across anonymized datasets at strict privacy levels (9.09% PR, 3.03% PR). Only parameters without scale transformation during the anonymization process are presented. Transformation and privacy models were applied as defined in the generic and Use case–specific scenario. Proportion 95% confidence intervals (95% CI) based on the Wilson score interval served to calculate 95% CI length overlap compared to the original dataset as proposed by Karr et al. [24]. Only affected variables are displayed. All estimates of the overall cohort were not affected (100% 95% CI overlap) and are not listed in the table. FD: female participants who were diabetic; FND: female participants who were non diabetic; MD: male participants who were diabetic; MI: myocardial infarction; MND: male participants who were non diabetic; MR: marketer risk; PR: prosecutor risk; PTA: percutaneous transluminal angioplasty; PTCA: percutaneous transluminal coronary angioplasty.

|  |  | Generic scenario | | | | | | | | Use case–specific scenario | | | | | | | |
| --- | --- | --- | --- | --- | --- | --- | --- | --- | --- | --- | --- | --- | --- | --- | --- | --- | --- |
|  |  | 9.09% PR | | | | 3.03% PR | | | | 9.09% PR | | | | 3.03% PR | | | |
|  |  | FND | FD | MND | MD | FND | FD | MND | MD | FND | FD | MND | MD | FND | FD | MND | MD |
|  | |  |  |  |  |  |  |  |  |  |  |  |  |  |  |  |  |
| Cardiovascular disease | | 83.0 | 85.1 | 90.4 | 86.4 | 67.6 | 89.3 | 93.9 | 55.7 | 80.5 | 88.6 | 88.7 | 92.0 | 56.7 | 78.8 | 87.0 | 56.2 |
| Arterial hypertension | | 86.7 | 98.0 | 90.0 | 97.1 | 90.0 | 100.0 | 90.0 | 84.9 | 86.7 | 100.0 | 95.0 | 93.8 | 72.2 | 95.8 | 95.0 | 97.1 |
| Cardiac valve replacement | | 96.2 | 91.8 | 94.4 | 100.0 | 96.2 | 94.8 | 97.4 | 96.4 | 92.9 | 93.2 | 97.4 | 100.0 | 90.0 | 91.8 | 97.4 | 96.4 |
| Coronary artery disease | | 90.4 | 82.1 | 95.4 | 82.2 | 76.4 | 89.1 | 97.3 | 61.4 | 84.0 | 92.0 | 89.1 | 87.7 | 65.0 | 84.1 | 88.1 | 62.2 |
|  | MI | 97.7 | 86.9 | 94.6 | 88.2 | 82.0 | 94.3 | 96.8 | 70.1 | 95.7 | 92.3 | 94.6 | 88.2 | 80.4 | 90.7 | 94.6 | 70.1 |
|  | Bypass surgery | 92.3 | 87.9 | 96.2 | 87.1 | 92.3 | 97.5 | 98.7 | 72.6 | 92.3 | 92.7 | 92.6 | 93.4 | 81.2 | 92.7 | 93.7 | 76.9 |
|  | PTCA | 89.0 | 88.6 | 99.0 | 88.9 | 80.2 | 91.7 | 98.0 | 75.1 | 80.2 | 96.7 | 90.0 | 92.1 | 67.2 | 91.0 | 91.1 | 71.9 |
| Cerebrovascular disease | | 86.8 | 91.3 | 93.8 | 92.9 | 72.9 | 95.1 | 96.3 | 76.0 | 83.0 | 94.2 | 93.8 | 96.4 | 71.8 | 92.2 | 96.3 | 77.2 |
|  | Stroke | 88.1 | 92.9 | 92.2 | 94.2 | 74.8 | 95.9 | 96.0 | 77.0 | 84.1 | 93.8 | 96.0 | 96.0 | 73.5 | 93.8 | 98.7 | 80.9 |
|  | Carotic surgery | 91.7 | 92.1 | 100.0 | 93.8 | 88.1 | 96.2 | 100.0 | 84.9 | 88.1 | 96.2 | 95.0 | 96.9 | 82.5 | 94.4 | 95.0 | 84.9 |
|  | Carotic intervention | 90.0 | 96.2 | 92.9 | 92.3 | 90.0 | 96.2 | 92.9 | 84.6 | 90.0 | 96.2 | 96.7 | 92.3 | 90.0 | 96.2 | 96.7 | 84.6 |
| Peripheral vascular disease | | 93.9 | 98.1 | 94.3 | 93.9 | 92.1 | 97.0 | 97.7 | 88.1 | 98.0 | 96.1 | 97.7 | 96.2 | 74.8 | 91.3 | 95.3 | 92.1 |
|  | Amputations | 89.8 | 96.2 | 95.1 | 93.3 | 96.7 | 98.1 | 98.4 | 93.3 | 89.8 | 100.0 | 95.1 | 93.3 | 87.1 | 98.1 | 98.4 | 90.4 |
|  | Y-graft | 94.4 | 96.2 | 93.8 | 100.0 | 90.0 | 96.2 | 93.8 | 100.0 | 100.0 | 96.2 | 96.9 | 88.9 | 82.6 | 96.2 | 96.9 | 100.0 |
|  | Other types of surgery | 94.1 | 92.0 | 97.8 | 96.9 | 94.1 | 100.0 | 95.5 | 96.9 | 96.9 | 93.4 | 100.0 | 100.0 | 83.5 | 96.8 | 100.0 | 100.0 |
|  | PTA | 96.9 | 92.9 | 98.5 | 97.2 | 90.4 | 94.0 | 96.9 | 94.4 | 96.9 | 96.4 | 95.4 | 97.2 | 79.4 | 89.5 | 96.9 | 94.4 |

**Table S10.** 95% CI overlap of the comparison of patients with diabetes mellitus (DM) with and without presumed diabetic nephropathy (DN) and patients without diabetes mellitus on estimate-level across anonymized datasets. Transformation and privacy models were applied as defined in the generic health data Use case–specific scenario. Proportion 95% confidence intervals (95% CI) based on the Wilson score interval served to calculate 95% CI length overlap compared to the original dataset as proposed by Karr et al. [24]. Only renal biopsy was affected in this table. All other variables (not shown) have 100% 95% CI overlap. DM w DN: diabetes mellitus with diabetic nephropathy; DM wo DN: diabetes mellitus without diabetic nephropathy; MR: marketer risk; No DM: no diabetes mellitus; PR: prosecutor risk.

| Renal biopsy | 50% PR + 9.09% MR | | | 50% PR + 3.03% MR | | | 9.09% PR | | | 3.03% PR | | |
| --- | --- | --- | --- | --- | --- | --- | --- | --- | --- | --- | --- | --- |
|  | DM w DN | DM wo DN | No DM | DM w DN | DM wo DN | No DM | DM w DN | DM wo DN | No DM | DM w DN | DM wo DN | No DM |
|  |  |  |  |  |  |  |  |  |  |  |  |  |
| Generic scenario | 100.0 | 75.3 | 70.8 | 100.0 | 78.7 | 86.2 | 93.1 | 70.2 | 61.6 | 91.3 | 78.7 | 15.4 |
| Use case–specific scenario | 91.3 | 87.2 | 84.4 | 96.6 | 97.9 | 93.8 | 91.3 | 51.6 | 56.2 | 84.2 | 28.3 | 0.0 |

**Figure S1.** Formation, dimensionality and volume of datasets. We selected variables for our real-world application scenario and pre-processed the data of the GCKD study. The curated dataset was composed of 70 variables and is referred to as the *original* dataset. The original dataset was anonymized in two different configuration set-ups. In both scenarios, suppression was limited to 10%. In the generic scenario, generalization hierarchies were defined according to generally applicable criteria in the health data context and no restrictions were applied in the generalization (standard set-up). In the Use case–specific scenario, we defined generalization hierarchies according to our real-world application and restricted generalization in relevant variables. To account for collinearity, we removed BMI in the generic scenario, and height and weight in the use case-specific one. We then reduced re-identification risk according to probabilistic PR and MR risk models. We used k-anonymity aiming at the PR and (k’,2)-strict-average risk capturing the combined PR and MR. We varied the risk threshold from 100% to 2% respectively. Technical realization was performed via ARX. The target function applied by ARX for determining the utility of differently anonymized datasets was configured to capture individual variable granularity. Dimensionality is described by the number of variables, volume by the number of records (n) including missing data. MR: marketer risk; PR: prosecutor risk.


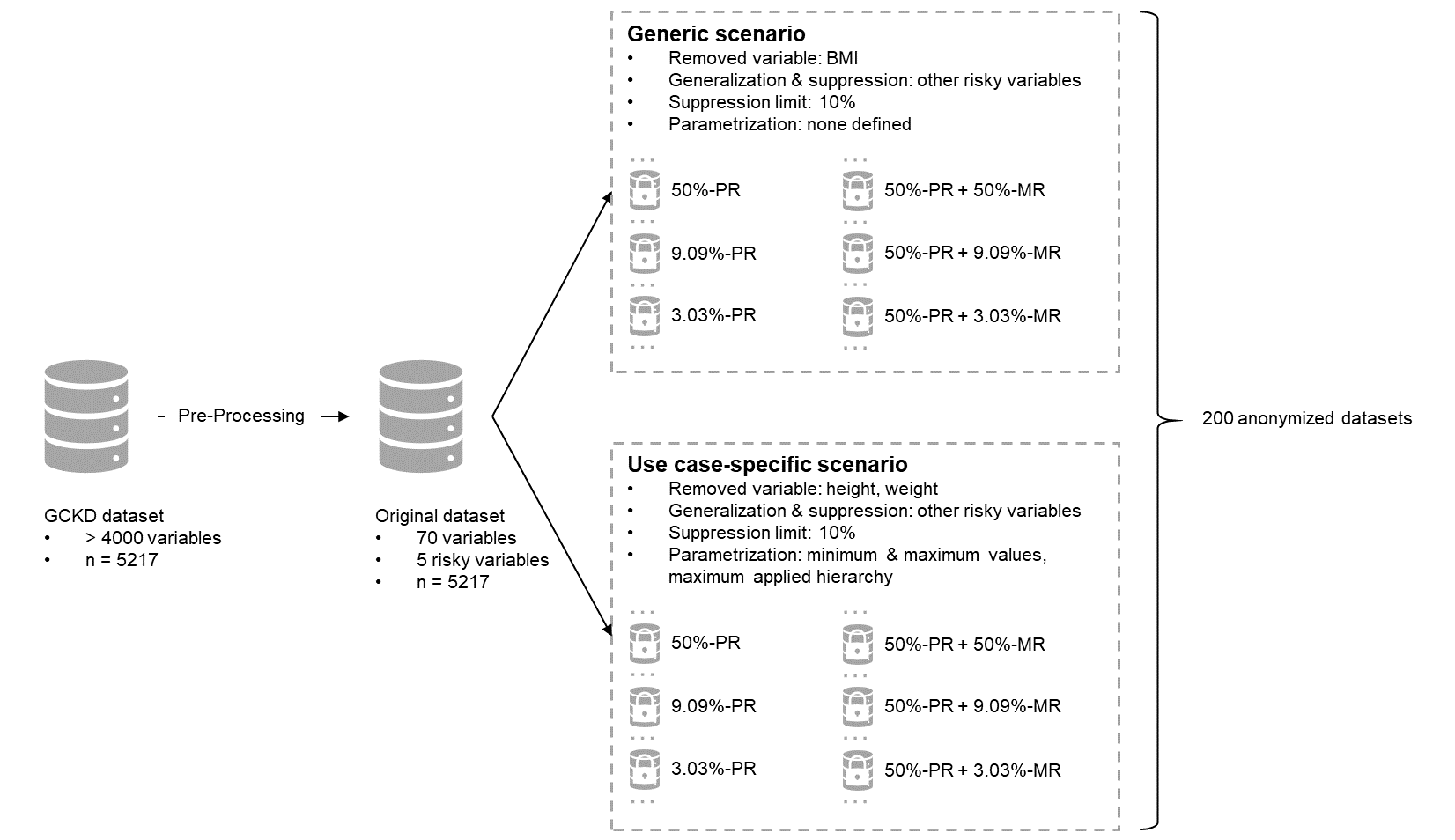


**Figure S2.** Privacy-utility curves based on general-purpose utility metrics. Granularity and non-uniform entropy served as general-purpose utility metrics. Privacy is demonstrated as 1 – empirical maximum PR, average PR (i.e., MR) and minimum PR. We used the anonymization processes implementing thresholds on 50%-PR + MR for generating the points on the curve: 50%-PR + 9.09 MR and 50%-PR + 3.03% MR. Results of granularity in the (A) generic and (C) use case–specific anonymized data sets and results of entropy in the (B) generic and (D) use case–specific anonymized data sets are shown. Note: the extreme points at (0,100) and (100,0) have been added to the graph and were not directly measured. MR: marketer risk; PR: prosecutor risk.


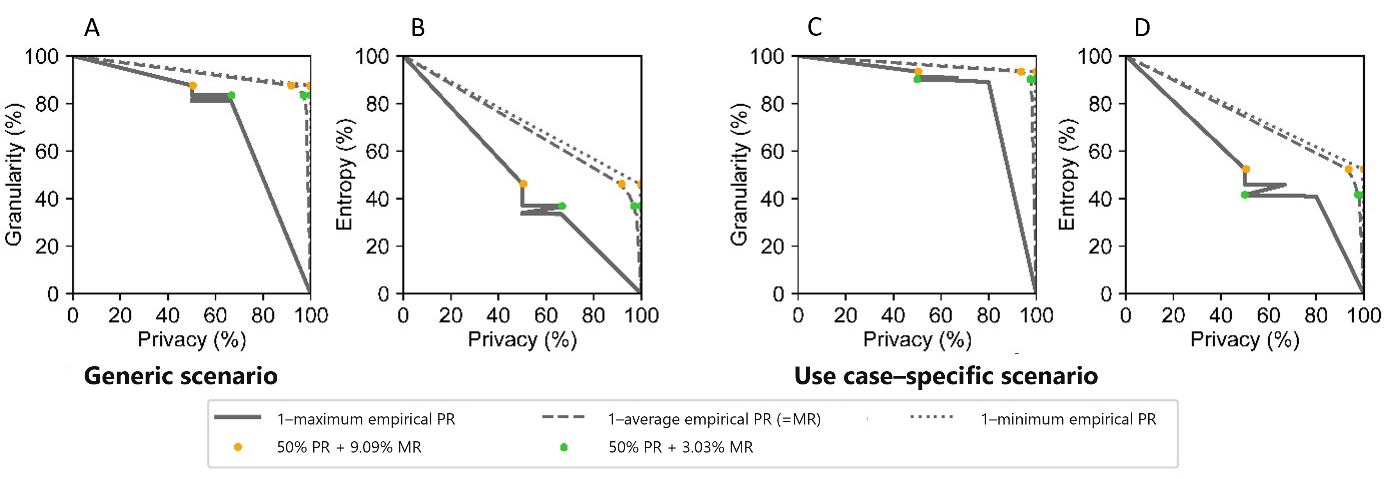


**Figure S3.** Privacy-utility curves using use case-specific utility metrics based on 95% CI overlap. CI overlap was calculated on dataset-level (overall 95% CI overlap) and analysis level. Two analyses (glomerular filtration rate and albuminuria categories and comparison of estimated glomerular filtration rate equations) were not affected by anonymization at all (100% overlap) and are therefore not displayed separately. Privacy is demonstrated as 1 – maximum PR, average PR (i.e., MR) and minimum PR. We used the anonymization processes implementing thresholds on 50%-PR + MR for generating the points on the curve: 50%-PR + 9.09 MR and 50%-PR + 3.03% MR. Results of the overall 95% CI overlap in the (A) generic and (G) use case–specific anonymized data sets and results of the 95% CI overlaps on analysis level in the (B-F) generic and (H-L) use case–specific anonymized data sets are shown. Results at the estimate level are shown in Tables S2-S10 in Multimedia Appendix 1. Note: the extreme points at (0,100) and (100,0) have been added to the graph and were not directly measured. MR: marketer risk; PR: prosecutor risk.


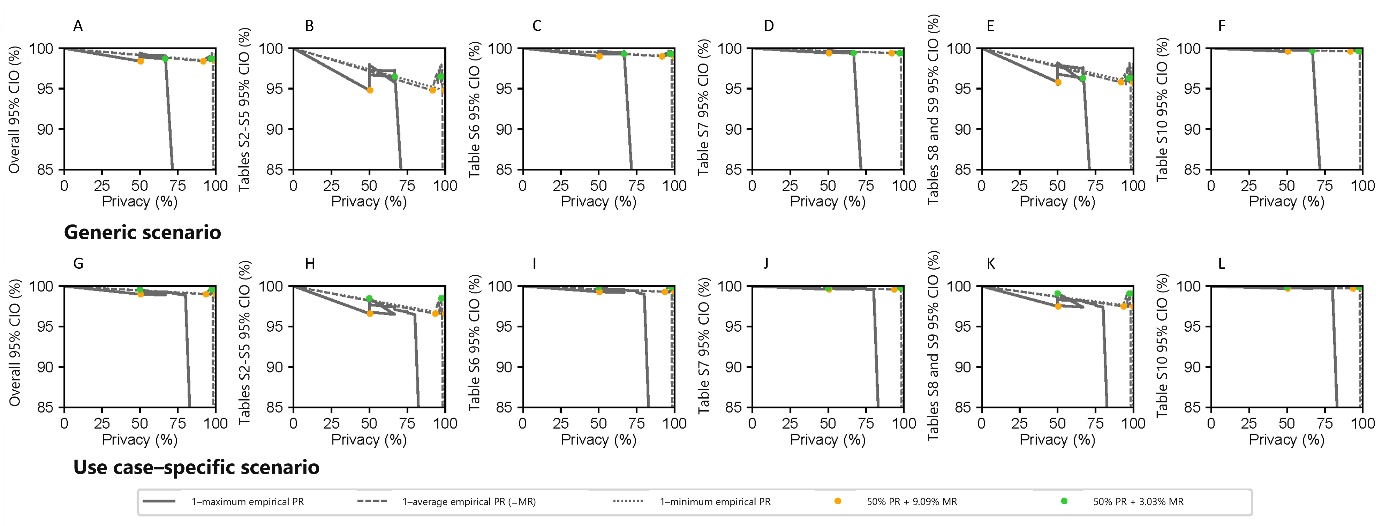


**Figure S4.** Intervals of calculated BMI in the generic scenario. In the generic scenario, BMI was calculated using the generalized data of height and weight. Intervals are indicated for the privacy levels 50% PR + 9.09% MR (A), 50% PR + 3.03% MR (B), 9.09% PR (C) and 3.03% PR (D). BMI: body mass index.


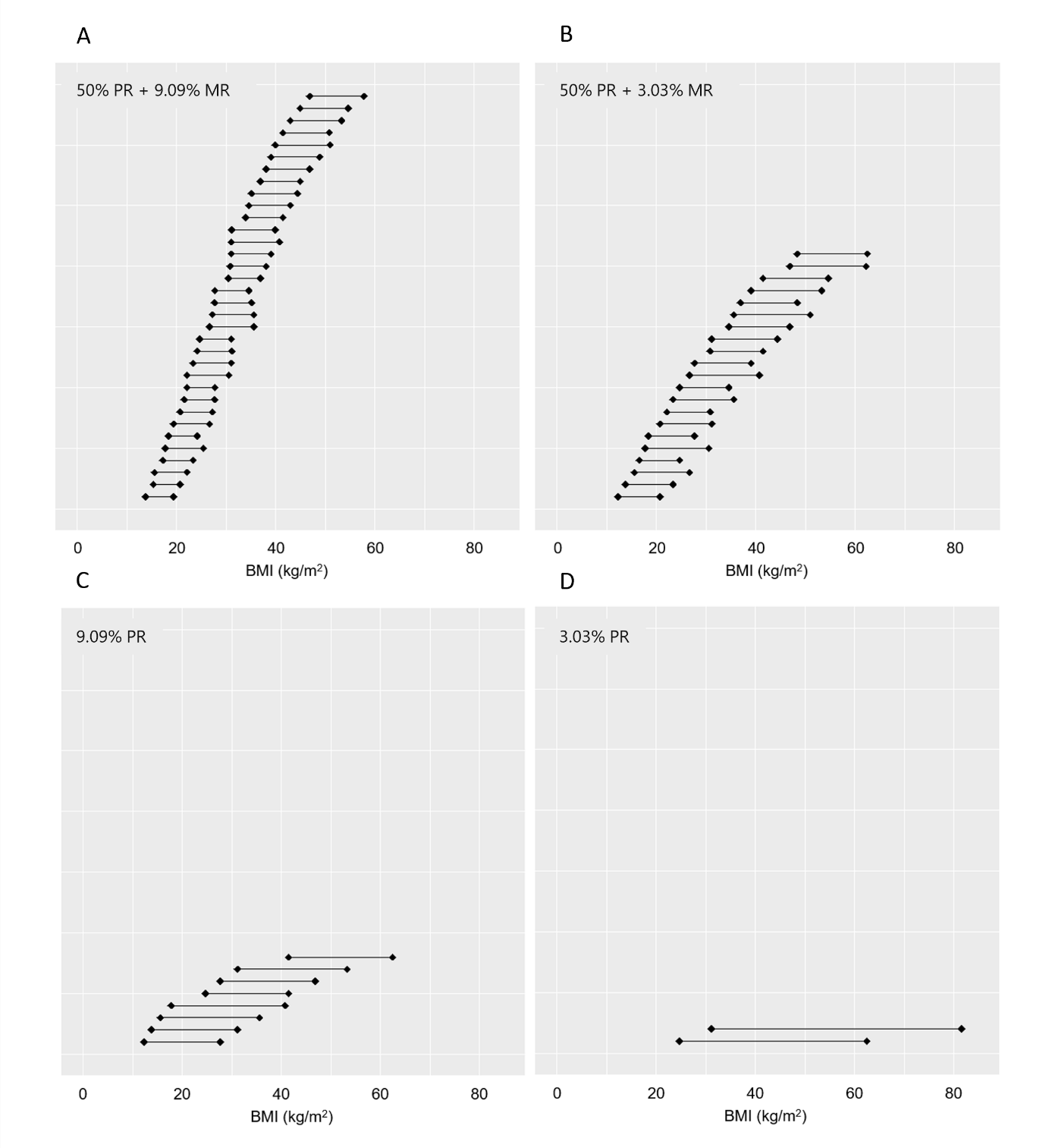


.

**Figure S5.** Illustration of height and weight of female non-diabetics in the original and anonymized datasets in the generic scenario. Bar plots illustrate counts for height (A) and weight (B) of anonymized data in the generic scenario. Data at privacy levels 50% PR + 9.09% MR, 50% PR + 3.03% MR, 9.09% PR and 3.03% PR is displayed, the original data illustrated as density plot in grey.


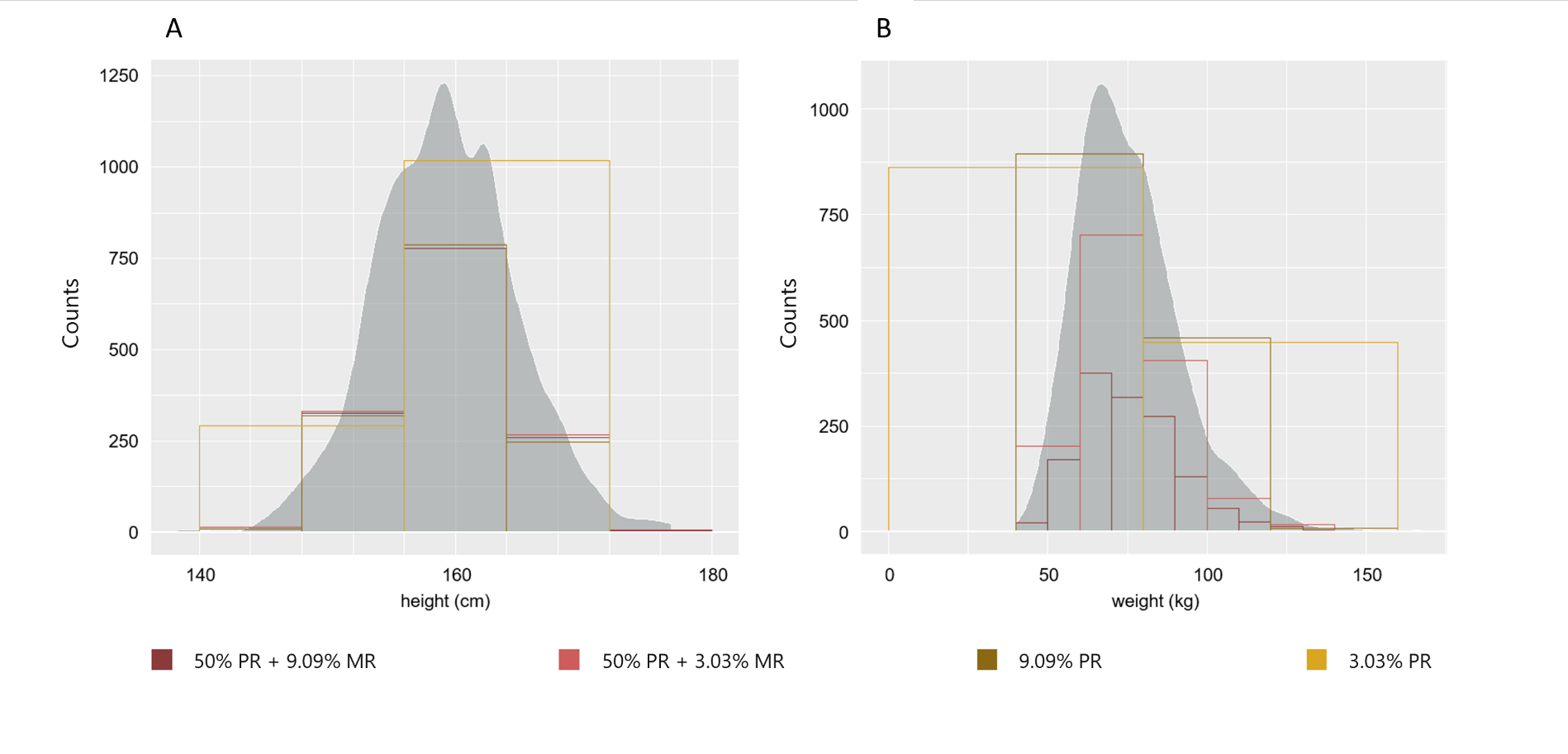

Supplement: Multimedia Appendix 1 [file jmir_v26i1e49445_app1.docx]
